# Competitive ELISA for a serologic test to detect dengue serotype-specific anti-NS1 IgGs using high-affinity UB-DNA aptamers

**DOI:** 10.1038/s41598-021-97339-8

**Published:** 2021-09-09

**Authors:** Ken-ichiro Matsunaga, Michiko Kimoto, Vanessa Weixun Lim, Tun-Linn Thein, Shawn Vasoo, Yee-Sin Leo, William Sun, Ichiro Hirao

**Affiliations:** 1 Institute of Bioengineering and Bioimaging, 31 Biopolis Way, The Nanos, #07-01, Singapore, 138669 Singapore; 2 National Centre for Infectious Diseases, 16 Jalan Tan Tock Seng, Singapore, 308442 Singapore; 3 Department of Infectious Diseases, Tan Tock Seng Hospital, 11 Jalan Tan Tock Seng, Singapore, 308433 Singapore; 4 Lee Kong Chian School of Medicine, Nanyang Technological University, 59 Nanyang Dr., Experimental Medicine Building, Singapore, 636921 Singapore; 5 Saw Swee Hock School of Public Health, National University of Singapore, 12 Science Drive 2, #10-01, Singapore, 117549 Singapore; 6 Department of Physiology, Yong Loo Lin School of Medicine, National University of Singapore, Singapore, 117593 Singapore

**Keywords:** Biological techniques, Diseases

## Abstract

Serologic tests to detect specific IgGs to antigens related to viral infections are urgently needed for diagnostics and therapeutics. We present a diagnostic method for serotype-specific IgG identification of dengue infection by a competitive enzyme-linked immunosorbent assay (ELISA), using high-affinity unnatural-base-containing DNA (UB-DNA) aptamers that recognize the four categorized serotypes. Using UB-DNA aptamers specific to each serotype of dengue NS1 proteins (DEN-NS1), we developed our aptamer–antibody sandwich ELISA for dengue diagnostics. Furthermore, IgGs highly specific to DEN-NS1 inhibited the serotype-specific NS1 detection, inspiring us to develop the competitive ELISA format for dengue serotype-specific IgG detection. Blood samples from Singaporean patients with primary or secondary dengue infections confirmed the highly specific IgG detection of this format, and the IgG production initially reflected the serotype of the past infection, rather than the recent infection. Using this dengue competitive ELISA format, cross-reactivity tests of 21 plasma samples from Singaporean Zika virus-infected patients revealed two distinct patterns: 8 lacked cross-reactivity, and 13 were positive with unique dengue serotype specificities, indicating previous dengue infection. This antigen-detection ELISA and antibody-detection competitive ELISA combination using the UB-DNA aptamers identifies both past and current viral infections and will facilitate specific medical care and vaccine development for infectious diseases.

## Introduction

The recent COVID-19 (Coronavirus Disease 2019) pandemic has highlighted the importance of serologic tests, for infectious disease diagnostics complementary to PCR and biomarker-detection tests^1–4^. Serologic tests enable the detection of viral-specific antibodies, mainly IgM and IgG, produced in the body by responses to current and past infections. In addition, such tests are useful for the diagnoses of infection, including surveys of disease transmission, infection spread, and acquired immunity, as well as evaluations of vaccine development. Most typical serologic tests are methods to detect antibodies by binding to viral-related antigens using lateral flow devices and the enzyme-linked immunosorbent assay (ELISA) or chemiluminescent immunoassay (CIA) format^5–8^. In these methods, the sensitivity and specificity of detection due to the cross-reactivity with other related diseases are one of the key issues, as they cause false-negative and false-positive test results. Indeed, diseases such as COVID-19 and dengue virus (DENV) infection present with similar symptoms in the tropical and subtropical countries^9,10^.

We now report a novel serologic test by a competitive ELISA format, using high-affinity unnatural-base-containing DNA (UB-DNA) aptamers for dengue diagnostics. Dengue is an arthropod-borne flavivirus with four main serological types (DEN1‒4). Worldwide DENV infections have been estimated at more than 390 million annually, and the recent dramatic and global increase in incidence suggests that approximately 40% of the world’s population is now at risk^11^. The symptoms range from mild in most cases to severe and occasionally fatal^12^. Infection with one DENV serotype provides long-term protection from re-infection with the same serotype, but may enhance the disease from a secondary heterotypic infection. A secondary DENV infection with a different serotype from the primary infection is the greatest risk factor for severe disease, such as Dengue Haemorrhagic Fever and Dengue Shock Syndrome. Antibody-dependent enhancement (ADE) is thought to be one of the mechanisms responsible for severe dengue^13–19^. The immune history is important for understanding subsequent disease risk and protection^20–27^, and thus the ability to identify the previous infected serotype is invaluable to study the pathogenesis. Quantitative diagnostic methods for serotype identification in previously infected patients will allow us to elucidate whether the sequence of the dengue serotype infection affects the severity of the disease.

Currently, there is no specific treatment for dengue, but early diagnostics prompt proper medical attention and reduce the risk of fatality. Dengvaxia, a dengue vaccine, was developed by Sanofi Pasteur^28,29^. However, analyses revealed that the vaccination of persons who have never been infected with dengue led to a higher risk of more severe symptoms when they became infected after the vaccination^30–34^. Consequently, the World Health Organization (WHO) advised the use of the vaccine only in people previously infected with dengue. The effectiveness of the vaccine could be different for people previously infected with different serotypes of dengue, and more research is needed to understand the underlying mechanisms. Therefore, the development of detection methods for not only current infection but also past infection, including the serotype identification and quantification, is an urgent worldwide task^31,34^.

**Figure 1:**
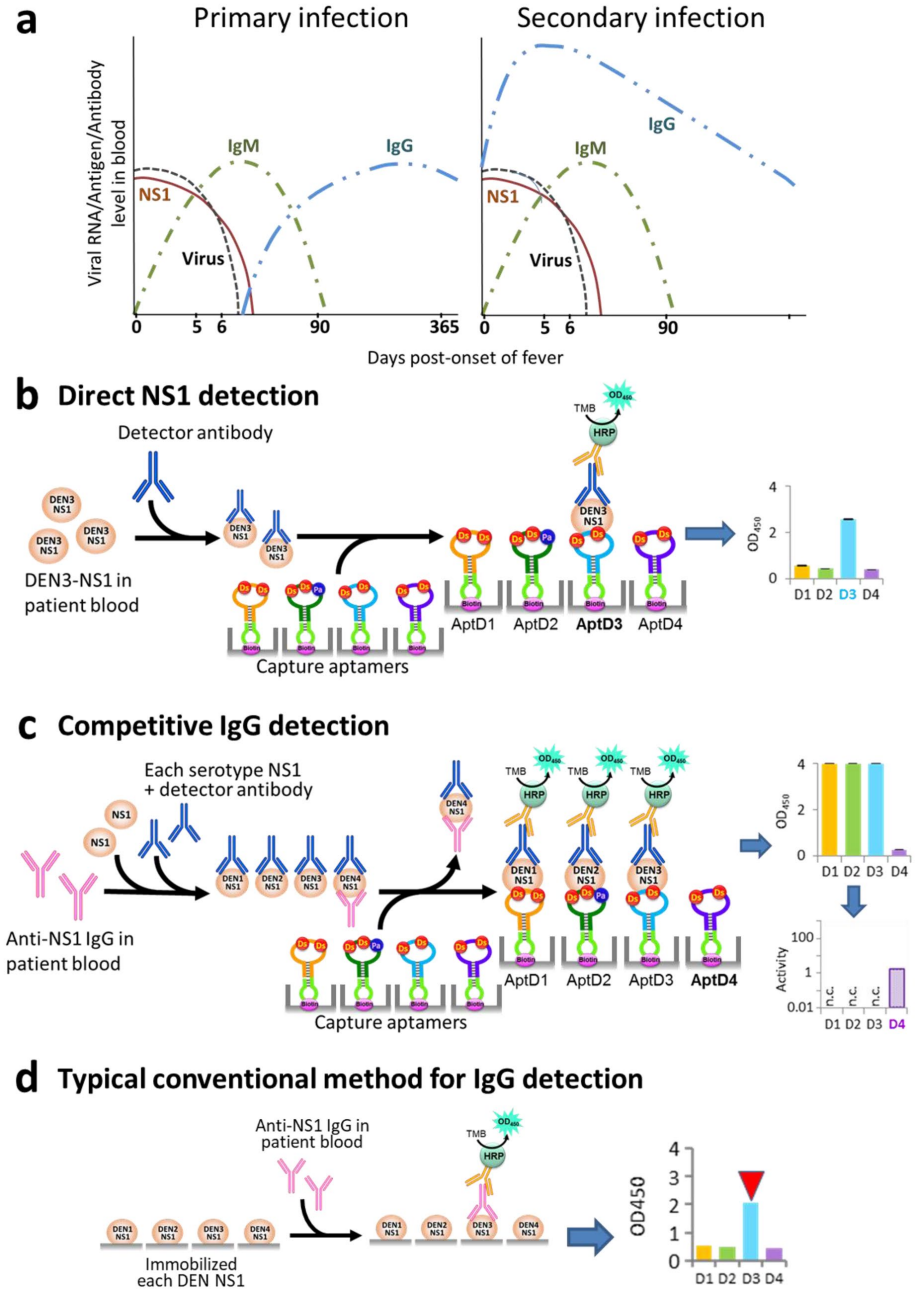
Dengue diagnostics using ELISA formats. **(a)** Schematic illustration of general detection sensitivity patterns for dengue viral (DENV) NS1 protein, virus (genome), and DENV-reactive IgM and IgG, in primary and secondary DENV infections^8,35^. **(b)** Direct DEN-NS1 detection in patient blood, by using our UB-DNA aptamers specific to each serotype NS1 protein as capture agents and an anti-DEN-NS1 antibody (Ab#D06) as a detector agent^35,49^. **(c)** Competitive IgG detection in patient blood, by modifying the DEN-NS1 detection method in **(b)**. If a blood sample contains IgGs binding to DEN-NS1, then the DEN-NS1 detection by UB-DNA aptamers is competitively inhibited. Thus, the addition of each serotype DEN-NS1 and UB-DNA aptamer to the blood sample, followed by mixing with the detector antibody, enables the detection and quantification of IgGs specific to each DEN-NS1 serotype. **(d)** Typical conventional method for IgG detection, using the antigen (NS1)-coated plates to capture anti-NS1 IgGs in a blood sample.

In the early stage (acute phase) of DENV infection (within one week after fever onset, Fig. 1a), the viral RNA can be identified by RT-qPCR. Virus-related materials, such as the envelope protein and non-structural protein 1 (NS1), can also be detected by ELISA or lateral flow assay (LFA), using antibodies to the antigens. RT-qPCR is useful for serotype identification in the early stage, but not the later stage, including convalescent phase^8,35,36^. For the serotype-specific NS1 detection in the early stage, several reports have described the generation of antibodies to each NS1 serotype^37–40^, but their diagnostic kits are not commercially available. In the later stage, the patients’ IgM and IgG antibodies to viral-related antigens, such as viral particles and NS1 proteins, are detectable by ELISA or LFA. The IgG production continues throughout life, and thus IgG detection enables the identification of past infections. However, the detection reliabilities are still limited, and the serotype identification remains difficult. Furthermore, the cross-reactivity of the tests with other infectious diseases showing similar symptoms, such as COVID-19 and other flavivirus infections, is a serious problem^9,10,41,42^. In some COVID-19 patients in Singapore, dengue serologic test kits were associated with false positivity for DENV infection^10^.

**Figure 2:**
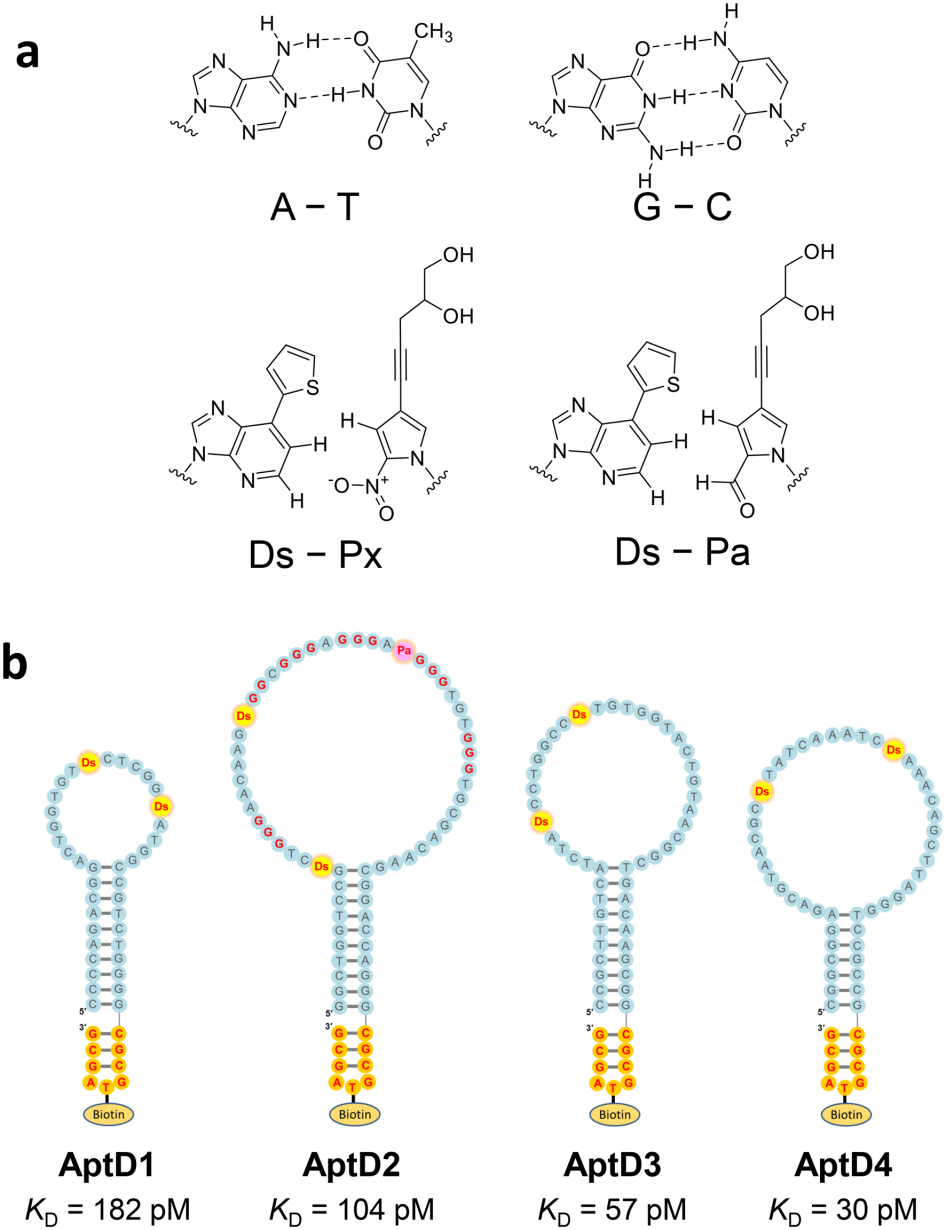
Natural and unnatural base pairs for UB-DNA aptamer generation and anti-DEN-NS1 UB-DNA aptamers. **(a)** Chemical structures of the natural base pairs, A–T and G–C, and the unnatural base pairs, Ds–Px and Ds–Pa. **(b)** Presumed secondary structures of UB-DNA aptamers that bind specifically to each serotype DEN-NS1: AptD1 to DEN1-NS1, AptD2 to DEN2-NS1, AptD3 to DEN3-NS1, and AptD4 to DEN4-NS1. Each aptamer’s dissociation constant (*K*_D_) determined by SPR analysis is shown. The unnatural Ds and Pa bases are shown in yellow and pink circles. At each aptamer’s 3′-terminus, the stable mini-hairpin sequence (CGCG(Bio-T)AGCG) is attached for stabilization against heat and nucleases and to introduce the biotinylated T (Bio-T) for aptamer immobilization.

Here, we present a simple and highly sensitive serotype-specific detection method for the anti-DEN-NS1 IgG antibodies in the early and later stages of DENV infection, as well as for the NS1 proteins in the early stage, by ELISA with high-affinity DNA aptamers (Fig. 1b,c). DNA aptamers are single-stranded DNA fragments that bind specifically to target molecules^43,44^, and are considered to serve as antibody alternatives. We developed a method (ExSELEX, genetic alphabet *Ex*pansion for *S*ystematic *E*volution of *L*igands by *EX*ponential enrichment) using an unnatural base pair (UBP), Ds–Px (Fig. 2a), to generate DNA aptamers with increased affinities to targets, in which unnatural base (UB) components, such as Ds and Px/Pa, are introduced into DNA aptamers as fifth and sixth letters^45–48^. Using ExSELEX, we generated a series of UB-DNA aptamers targeting each serotype or sub-serotype of DEN-NS1 proteins (Fig. 2b)^49^. The ELISA format using a sandwich system with these UB-DNA aptamers and an anti-DEN-NS1 antibody detects and identifies the serotype or sub-serotype (mutant variants in each serotype) of DEN-NS1 in clinical blood samples with extremely high specificity, in which these aptamers recognize a few amino-acid changes in each DEN-NS1 serotype (Fig. 1b)^49^. During the study, we serendipitously found that the UB-DNA aptamer binding to DEN-NS1 is serotype-specifically inhibited when anti-DEN-NS1 IgG antibodies were present in patient blood samples. Based on this observation, we developed a competitive ELISA format for a serological test to quantitatively detect serotype-specific IgGs for dengue diagnostics (Fig. 1c). Using this method, we analyzed clinical samples from dengue patients. The tests with the secondary infection samples revealed that the IgG production reacted more to the serotype of the past infection, rather than that of the secondary infection. We also examined the cross-reactivity of this method with IgGs in blood samples from Zika virus patients and COVID-19 vaccinated subjects. This dengue diagnostic system using the competitive ELISA format will provide valuable information about dengue epidemiology and pathogenesis, as well as vaccine usage^31,34^ and development^50,51^, and could be adapted for other diseases.

## Results

### UB-DNA aptamers targeting each DEN-NS1 serotype and the ELISA format

We previously developed an ELISA format of aptamer–antibody sandwich systems (Fig. 1b), using UB-DNA aptamers targeting each serotype or sub-serotype of DEN-NS1 (Supplementary Table 1 and Fig. 2), for diagnostics in the early stage of dengue infection (Fig. 1a)^49^. These high-affinity UB-DNA aptamers (*K*_D_ = 30 − 182 pM) were generated from five-letter Ds-DNA libraries by ExSELEX, involving the Ds −Px pair as a third base pair in PCR. In the aptamer generation, we used each DEN-NS1 serotype purchased from The Native Antigen Company. Three aptamers, AptD1, AptD3, and AptD4 targeting DEN1-NS1, DEN3-NS1, and DEN4-NS1, respectively, contained two Ds bases, while the isolated aptamer, AptD2, exhibiting the highest affinity to DEN2-NS1, contained two Ds and one Px bases. These three UBs in AptD2 are essential for the tight binding to DEN2-NS1. This Px base was incorporated into the aptamer by a mutation during PCR amplification of the DNA libraries in ExSELEX. Since the Px nucleoside is unstable and thus not suitable for chemical DNA synthesis, instead of the Px nucleoside, we used the Pa nucleoside, in which the nitro group is replaced with the aldehyde group (Fig. 2a)^49,52,53^, for the chemical synthesis of AptD2. Each of these four aptamers contains an extraordinarily stable mini-hairpin sequence^54–56^ at the 3′-terminus, in which the loop region is useful as a modification site with biotin for aptamer immobilization^48,57–59^.

For the ELISA format with aptamer and antibody sandwich systems, we employed an anti-DEN-NS1 monoclonal antibody (Ab#D06), which binds to all four serotypes of DEN-NS1 with 27–107 pM *K*_D_ values^49^. In the ELISA format, the mixtures of DEN-NS1 samples and the antibody Ab#D06 were incubated on an aptamer-immobilized plate, and then the signal was detected by the colorimetric output, using a secondary anti-IgG HRP-conjugated antibody. We determined the limits of detection (LOD) in buffer, which were 1.19–2.36 ng/mL for the DEN-NS1 samples purchased from The Native Antigen Company^49^.

### Development of serotype-specific anti-DEN-NS1 IgG detection by a competitive ELISA format

**Figure 3:**
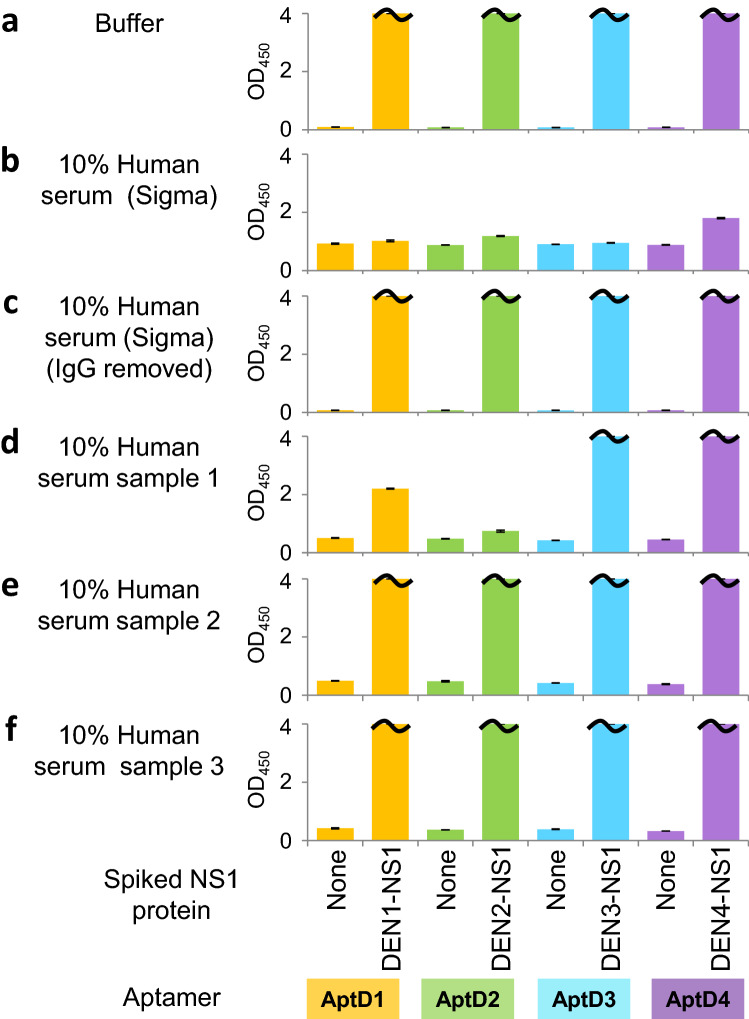
Inhibitory effects of human serum against direct NS1 detection. Inhibitory effects of different human serum samples were analyzed using the aptamer/antibody (Apt/Ab) ELISA format. Each UB-DNA aptamer was employed as the capture agent, and the antibody, Ab#D06, was used as the primary detector agent. Each serotype DEN-NS1 was added in buffer **(a)** or human serum purchased from Sigma (untreated in **b** or treated with protein A resin for removal of IgGs in **c**), or each human serum sample obtained from three different persons with no current dengue infection **(d–f)**. The mixtures were subjected to ELISA at the final 10% human serum concentration. A certain amount of the recombinant DEN-NS1 purchased from The Native Antigen Company (350 pg for DEN1-NS1, 350 pg for DEN2-NS1, 450 pg for DEN3-NS1, and 200 pg for DEN4-NS1) was added in each well (50 μL).

We next tested this ELISA format in the presence of human serum purchased from Sigma-Aldrich, for the direct detection of each DEN-NS1 from The Native Antigen Company (Fig. 3). Unexpectedly, we found that the NS1 detection was significantly inhibited in the serum (Fig. 3b), relative to that in buffer (Fig. 3a). One of the plausible causes is the presence of anti-DEN-NS1 IgG antibodies in the serum, which inhibited the binding of the aptamer to the additional NS1 proteins. To validate this IgG contamination theory, we removed the total IgG antibodies from the serum by treating it with protein A-immobilized resin, and confirmed the absence of inhibition with the treated serum (Fig. 3c). We also performed an ELISA using a serum sample from a Singaporean healthy volunteer who was currently not infected with dengue, to determine whether the serum inhibited the detection. Interestingly, the serum showed the serotype-specific inhibitions in the detection of DEN2-NS1, as well as DEN1-NS1 to some extent (Fig. 3d), suggesting that the person might have previously been infected with the dengue serotype 2 and/or serotype 1 viruses. Therefore, we obtained two other serum samples from healthy Japanese volunteers (PD0-1 and PD0-2) who have never been infected by DENV and performed an ELISA. As expected, the two serum samples did not inhibit the DEN-NS1 detection in our ELISA format (Fig. 3e,f). For further studies, we used these serum samples as a control without anti-DEN-NS1 IgGs. These results inspired us to develop a new method using the competitive ELISA format with each DEN-NS1 serotype sandwiched with each aptamer and Ab#D06, for the serotype-specific detection of anti-DEN-NS1 IgG antibodies in human serum samples (Fig. 1c).

For the serotype-specific IgG detection, we developed a simple quantification method for the anti-DEN-NS1 IgG activities (Supplementary Fig. 1). To this end, we performed competitive-inhibition in ELISA using a series of different volumes (0.05, 0.1, 0.2, 0.5, and 5 μL) of patient serum, in the presence of a certain amount of each DEN-NS1 serotype (The Native Antigen Company). After the absorbance measurement at 450 nm (OD_450_) in the ELISA format, the OD_450_ values were plotted against the volume of serum, and we calculated the serum volume required to give an OD_450_ of 1.0. We then defined the relative IgG activity (Activity), by the following formula: Activity = 5/(the serum volume required for an OD_450_ of 1.0). To equalize the sensitivities of the DEN-NS1 detection among the four serotypes, we adjusted the amounts of immobilized aptamers and spiked DEN-NS1 for each serotype (Supplementary Fig. 2).

### Serotype-specific detection of anti-DEN-NS1 IgG antibodies in patient samples

**Figure 4:**
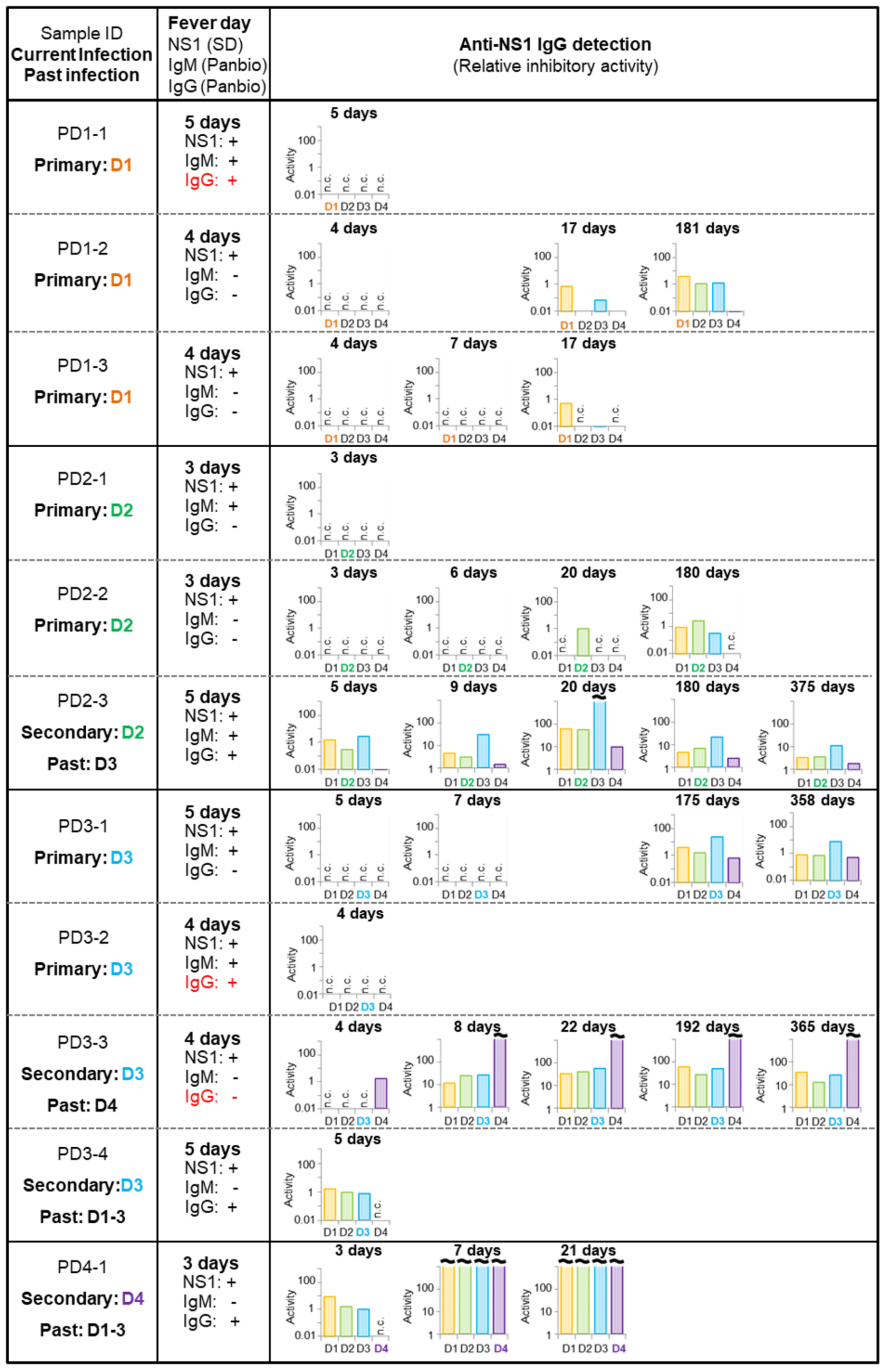
Anti-DEN-NS1 IgG detection in Singaporean clinical samples using the competitive Apt/Ab ELISA format. The clinical human serum or plasma samples (PD1-1 to PD4-1) were obtained in 2016 in Singapore. The direct DEN-NS1 detection of the samples at the early stage (3–5 days after fever onset) was performed by the SD BIOLINE Dengue NS1 Ag rapid test. The IgM and IgG detections were performed with a Panbio Dengue Duo Cassette. The quantitative IgG activities to each serotype DEN-NS1 in the clinical samples were determined by our competitive Apt/Ab ELISA format, which also identified the current infection status (primary or secondary) and the serotype of the past infection (past). The discrepancies with the IgG detection between the Panbio LFA and our competitive ELISA data are indicated in red. The RT-PCR analysis of the samples at the early stage also confirmed the DENV serotype caused by the current infection.

Using this competitive ELISA format and the IgG quantification method, we measured the longitudinal changes in the IgG production at the acute and convalescent phases and identified the serotype specificities of the archived blood samples from eleven Singaporean patients (PD1-1 to PD4-1) with acute DENV infection (Fig. 4 and Supplementary Figs. 3–6). The dengue serotype of the current infection in each sample was also identified by our antigen-detection ELISA and an FTD dengue differentiation RT-qPCR test from Fast Track Diagnostics, and by sequencing the RT-PCR amplicons of the clinical samples obtained within 3‒5 days after fever onset^49^. In addition, we performed the DEN-NS1, IgM, and IgG detections in these samples with the commercially available LFA kits from SD BIOLINE for DEN-NS1 detection and from Panbio for IgM/IgG detection.

Our competitive ELISA format successfully detected the IgGs even after one year in the recovered patients, as shown in the samples PD2-3, PD3-1, and PD3-3. The samples can be categorized into two groups by the IgG detection: one group included PD1-1, PD1-2, PD1-3, PD2-1, PD2-2, PD3-1, and PD3-2, in which the IgG was not detected within a week after fever onset, and the other group included PD2-3, PD3-3, PD3-4, and PD4-1, in which the IgG was detected within 3‒5 days after fever onset (acute phase). These data suggested that the latter patients were previously infected by dengue (Fig. 1a). Thus, the first group most likely represented the primary infection, and the second group was a secondary or higher infection.

There were some discrepancies in PD1-1, PD3-2, and PD3-3 between our IgG detection and the conventional LFA method for IgM/IgG detection. The visual judgement using the LFA format was often ambiguous, and all of the longitudinal IgG detection data supported the higher accuracy of our method over that of the LFA format. The sensitivity and specificity of the LFA kit reported in the literature are 71.9% and 95%, respectively^8^. Thus, our IgG detection might faithfully identify the primary or secondary infection of patients within 3‒5 days after fever onset.

Our data indicate that the inhibition of the aptamer binding to each DEN-NS1 serotype resulted from the anti-NS1 IgG, rather than the anti-NS1 IgM. No inhibition of the DEN-NS1 detection in our competitive ELISA occurred within the primary infection samples in the first week after fever onset, and the inhibition became detectable at 17 days or thereafter in longitudinal studies. In the samples from the patients with the primary infection, IgM was detected in PD1-1, PD2-1, PD3-1, and PD3-2 in the early phase (3–5 days) by the Panbio test (Fig. 4). Although the Panbio test detects IgMs targeting DENV envelope proteins, IgMs to DEN-NS1 proteins might also be present in these samples. However, the majority of the IgM in the acute phase of DENV infection reportedly targets viral particles or envelope proteins^60^, and the low abundance of anti-NS1-IgMs might reduce the sensitivity of the NS1-specific IgMs. To confirm the IgG-specific detection of our competitive ELISA format, further examinations using a large number of DENV clinical samples will be required.

Using the four samples (PD1-2, PD1-3, PD2-2, and PD3-1) collected 17 or more days after fever onset in the primary infection, our competitive ELISA format identified the serotype of the current infection from the highest serotype-specific activity of each detected IgG. Our results were identical with those obtained by RT-qPCR and sequencing^49^. In the samples obtained six months later (PD1-2 and PD2-2), our data indicated that the serotype-specificity of IgGs broadened to other serotypes.

In the secondary infection samples (PD2-3, PD3-3, PD3-4, and PD4-1), the initial IgG level was not identical to the serotype-specificity of IgGs in the current infection, which might mainly reflect the serotypes of past infection^26,27,61^. Even after one week, the production of the IgG antibodies that predominantly recognized the serotype resulting from the presumable past infection increased sharply, as compared to the IgGs produced from the current secondary infection. Although the predominance of the past infection varied depending on the patients, the PD2-3 and PD3-3 patient samples revealed the massive production of the IgG antibodies to the past serotype infection, and thus the competitive ELISA format might identify the dengue serotype of the past infection.

### Comparison with the typical conventional serologic test

To evaluate the competitive ELISA format, we compared the specificity and sensitivity of the format with a conventional serologic test reported in the literature^50,62^. There are several available kits for the direct DENV IgM and IgG detections by ELISA formats, which cannot identify the serotype, and their sensitivities and specificities for IgG detection are 45–56% and 93–95%, respectively, with Panbio Dengue Virus IgG Capture ELISA, and 55–89% and 64–99% with SD BIOLINE Dengue IgG ELISA^8,63,64^. Thus, we performed the conventional serologic test by incubating clinical samples on the plate immobilized with each serotype DEN-NS1 (The Native Antigen Company), followed by detection with a secondary anti-IgG HRP-conjugated antibody (Fig. 1d).

**Figure 5:**
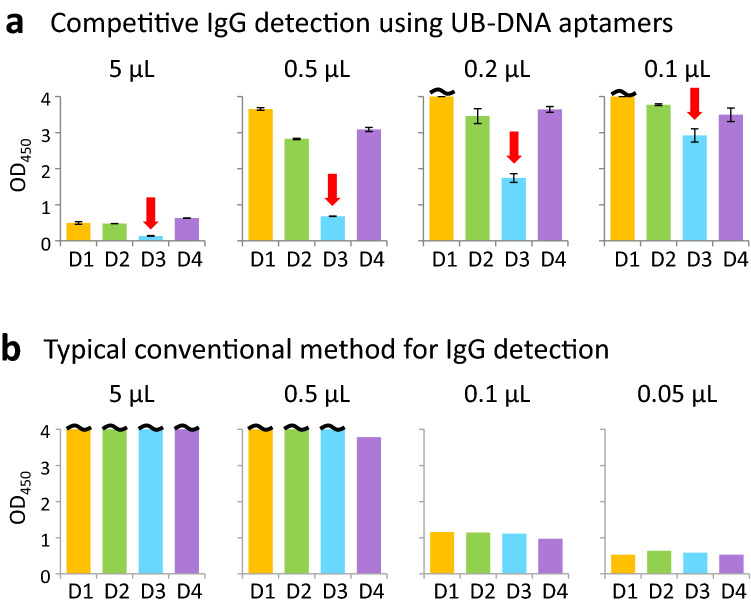
Comparison of the serotype-specific IgG detection by the competitive and conventional ELISA formats using a clinical sample (plasma, PD2-4). **(a)** The competitive Apt/Ab ELISA format (Fig. 1c), displaying the inhibition of the aptamer–DEN-NS1 binding by IgGs in our competitive Apt/Ab ELISA format using 0.1 to 5 µL of a clinical sample obtained within 4 days after fever onset. The amounts of the recombinant DEN-NS1 (The Native Antigen Company) added to each well (50 μL) were 350 pg for DEN1-NS1, 350 pg for DEN2-NS1, 450 pg for DEN3-NS1, and 200 pg for DEN4-NS1. Red arrows indicate the higher inhibition of the spiked DEN-NS1 detection among the four serotypes of DEN-NS1, suggesting that the IgG in the sample is more specific to DEN3-NS1. The results revealed that the current infection of PD2-4 might be secondary, and the past infection might be DENV serotype 3, although the Panbio LFA kit did not detect IgGs from the sample (Supplementary Table 2). **(b)** Direct IgG detection by a typical conventional ELISA method (Fig. 1d) using 0.05–5 μL of the sample. Each well was coated with 50 ng recombinant DEN-NS1 protein.

First, we compared the inhibition specificity by the competitive ELISA (Fig. 5a) and the IgG detection by the conventional method (Fig. 5b), using various volumes (0.05–5 μL) of a Singaporean clinical plasma sample, PD2-4. Although the IgGs in the sample were not detected by the LFA kit, our method and the conventional methods clearly detected anti-DEN-NS1 IgGs. In our competitive ELISA, the aptamer binding to serotype 3 of DEN-NS1 (DEN3-NS1) was mainly inhibited with the clinical sample (0.1–5 μL of PD2-4), indicating that the IgGs in the sample specifically bind to DEN3-NS1. However, in the conventional method, the IgGs equally bound to all four DEN-NS1 serotypes and the serotype-specificity of the IgGs was unobservable.

**Figure 6:**
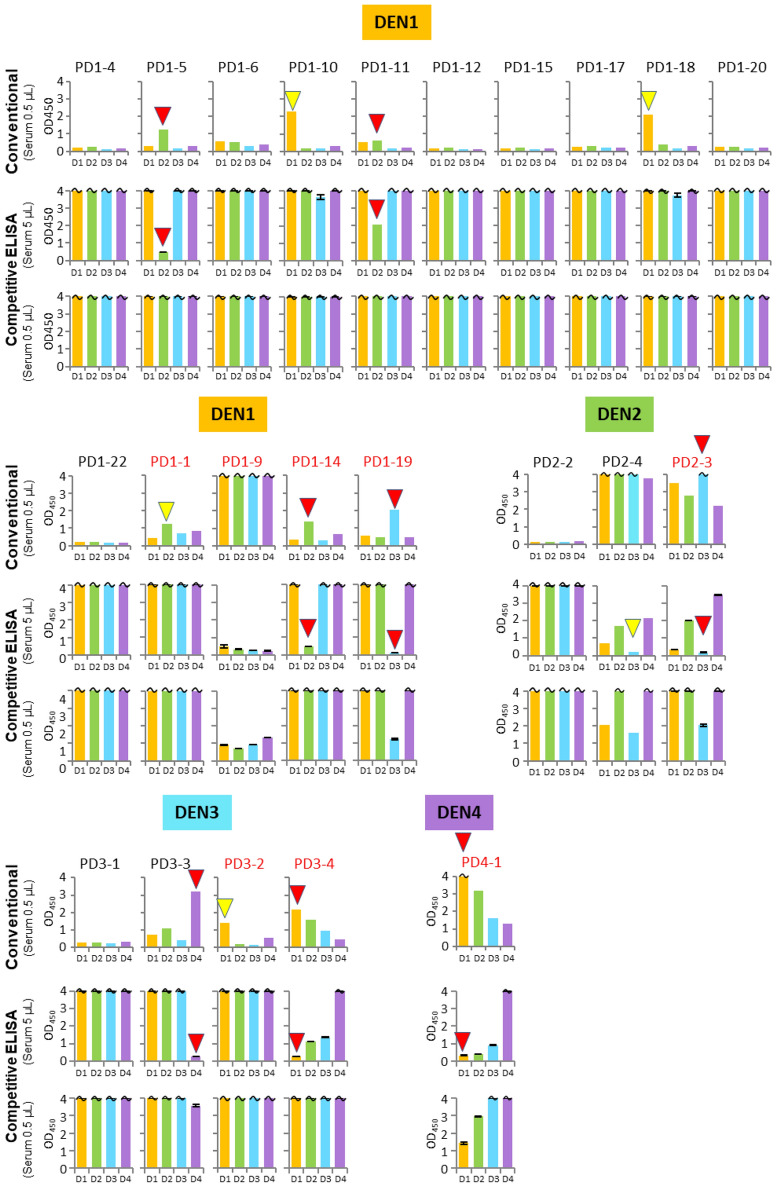
Comparison of the IgG detection sensitivity and selectivity using various clinical samples with two types of ELISA. Conventional: the direct IgG detection by the typical ELISA method in Figs. 1d and 5b using 0.5 µL of the serum or plasma samples. Competitive ELISA: the aptamer–DEN-NS1 binding inhibition by our competitive Apt/Ab ELISA (Figs. 1c and 5a) using 0.5 µL and 5 µL of the serum or plasma samples. The amounts of the spiked DEN-NS1 are indicated in Supplementary Fig. 1. The similar patterns of the serotype-specific IgG detection by both ELISA formats are indicated by red triangles. IgG detection patterns that differ between the conventional and competitive ELISA formats are indicated by yellow triangles. The patient sample names (indicated in red) were assigned as secondary infection by the LFA test.

Next, we extensively tested the IgG detections using 23 Singaporean clinical samples (PD1-1 to PD4-1) obtained within 3–5 days after fever onset (Supplementary Table 2). The serotypes of the current infection of each sample were initially determined by RT-qPCR and sequencing^49^, and the IgM and IgG detections were also performed with the LFA Panbio kit. The LFA tests detected anti-DEN-NS1 IgGs in the PD1-1, 1-9, 1-14, 1-19, 2-3, 3-2, 3-4, and 4-1 samples, suggesting that these patients had secondary infections. However, there are some discrepancies (including PD2-4, as shown in Fig. 5) among the results obtained using the LFA Panbio kit and our conventional and competitive ELISA methods (Fig. 6). The conventional and/or competitive ELISA methods detected IgGs in the samples of PD1-5, 1-10, 1-11, 1-18, 2-4, and 3-2, which were not detected by the LFA Panbio kit. In addition, the PD1-1, 1-10, 1-18, and 3-2 samples were IgG-positive by the conventional method, but the competitive ELISA detected no IgGs in these samples. Therefore, further extensive research is necessary to assess the sensitivity and specificity of our method by comparisons to those of the conventional method, using well-characterized samples before and after DENV infection.

### Comparison of the competitive ELISA formats between aptamer–antibody and antibody–antibody sandwich systems

**Figure 7:**
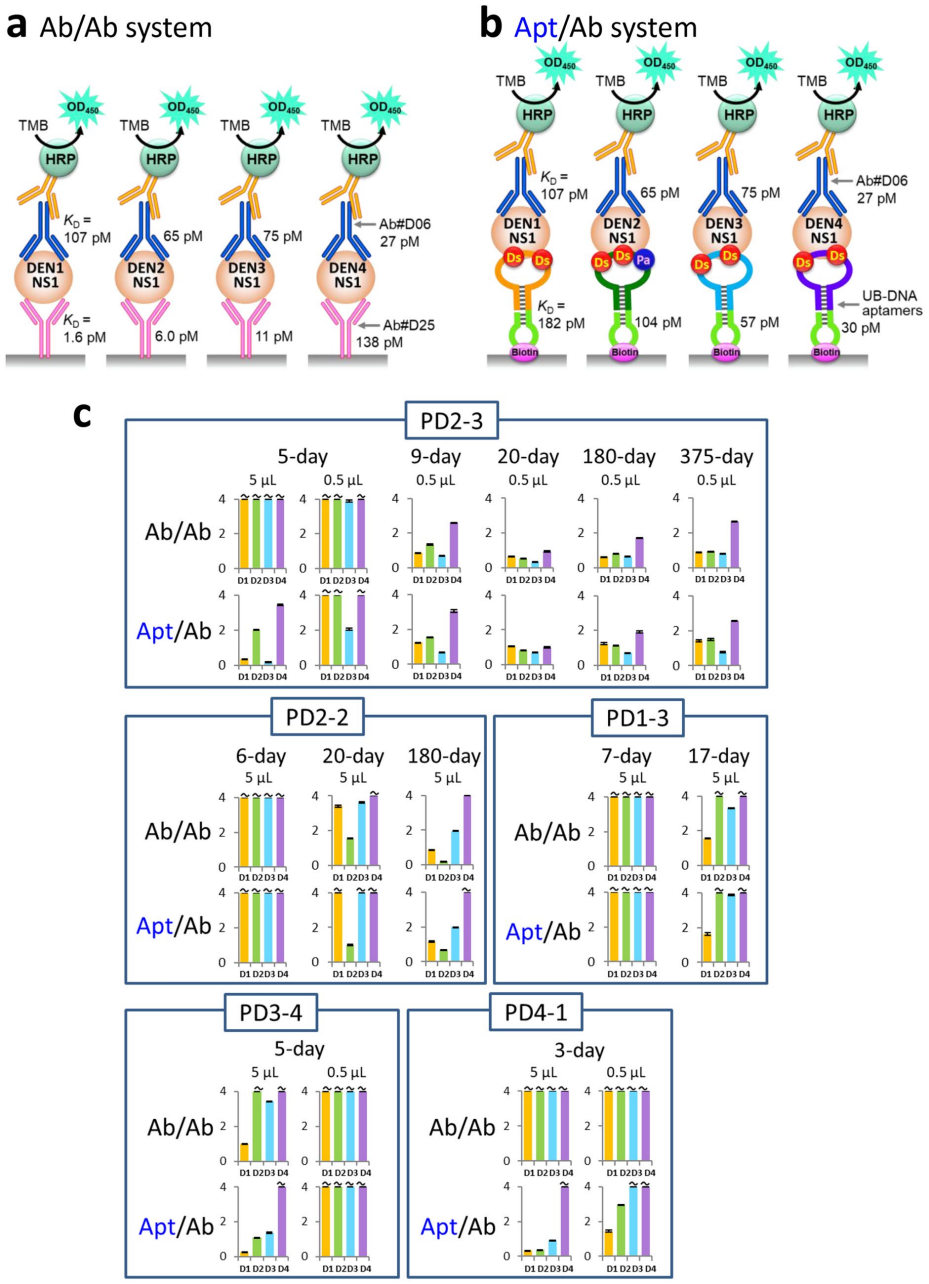
Comparison of the competitive ELISA formats between aptamer–antibody (Apt/Ab) and antibody–antibody (Ab/Ab) sandwich systems. **(a,b)** Schematic representation of the components in each system, using four DEN-NS1 serotypes. In the Ab/Ab system, the same antibodies for the capture and detection reagents were used. In the Apt/Ab system, each UB-DNA aptamer was used for each DEN-NS1 serotype. **(c)** Comparison of ELISA signal patterns (inhibition) of each clinical sample in different time courses.

Competitive or blocking ELISA formats using the inhibition between protein–protein or protein–antibody interactions are well-known methods^6,65,66^. Theoretically, the DENV-serotype-specific IgG detection by the competitive ELISA format is also possible by using an antibody–antibody sandwich system, even if these antibodies have no specificity to each DEN-NS1 serotype (Fig. 7a)^67^. We tested this antibody–antibody system using Ab#D06 and Ab#D25, which do not compete with each other for the DEN-NS1 binding, and compared the longitudinal changes of the IgG detections with the aptamer–antibody system in the PD1-3, 2-2, 2-3, 3-4, and 4-1 samples (Fig. 7 and Supplementary Figs. 3–6). The anti-DEN-NS1 IgG in the patient serum also inhibited the DEN-NS1 ternary complex formation with the antibody‒antibody sandwich pair, and the test exhibited similar patterns to those obtained by the aptamer‒antibody sandwich pair. However, the antibody‒antibody pair could not detect the IgG activities in the early stage of infection, such as the day 5 sample of PD2-3 and the day 3 sample of PD4-1.

One of the plausible explanations for the difference in the sensitivities between the antibody–antibody and aptamer–antibody systems is the variations in the affinities of antibodies and aptamers to each DEN-NS1 protein serotype. We used high-affinity anti-DEN-NS1 antibodies for the competitive ELISA. The *K*_D_ values of Ab#D06 (detector agent) to DEN1-NS1, DEN2-NS1, DEN3-NS1, and DEN4-NS1 are 107, 65, 75, and 27 pM, respectively, and those of Ab#D25 to each DEN-NS1 are 1.6, 5.0, 11, and 138 pM, respectively^49^ (Fig. 7a). Ab#D25 tightly binds to DEN1-NS1, DEN2-NS1, and DEN3-NS1 and might reduce the inhibitory effect of the anti-DEN-NS1 antibodies, as shown in the negative results in the acute phase of the clinical samples PD2-3, PD3-4, and PD4-1, in Fig. 7c. As compared to Ab#D25, the *K*_D_ values of the aptamers are less biased to each DEN-NS1 serotype (*K*_D_ = 182 pM for AptD1 to DEN1-NS1, 104 pM for AptD2 to DEN2-NS1, 57 pM for AptD3 to DEN3-NS1, and 30 pM for AptD4 to DEN4-NS1)^49^. To identify the serotype specificities of anti-DEN-NS1 IgGs, the ideal competitive ELISA would require the unbiased affinities of antibodies and aptamers against each DEN-NS1 serotype.

### Cross-reactivity of the competitive ELISA format using the aptamer-antibody system with blood samples from Zika virus-infected patients and COVID-19 vaccinated volunteers

For practical use, our competitive ELISA format requires further validation, such as the identification of cross-reactivities with other related infections and vaccinations. To this end, cohort follow-up studies could determine the sensitivity and specificity of our method. However, the issue is the collection of well-characterized clinical samples without and with DENV infection, including serotype information, because of the possibility of multiple, unrealized past infections. Since the first DENV infection is rarely severe, patients are often unaware of the infection or attribute the symptoms to a common cold. In addition, other cross-reactive viral infections, such as Zika virus (ZIKV), complicate the validation as exemplified by dengue and Zika co-infection cases in Singapore^68^. However, several reports have indicated the high specificities of antibodies against NS1 proteins between dengue and Zika viruses, as compared to the antibody cross-reactivities of envelope proteins among flaviviruses^20,69–73^. Thus, we examined the cross-reactivity of our method with Zika patient samples.

**Figure 8:**
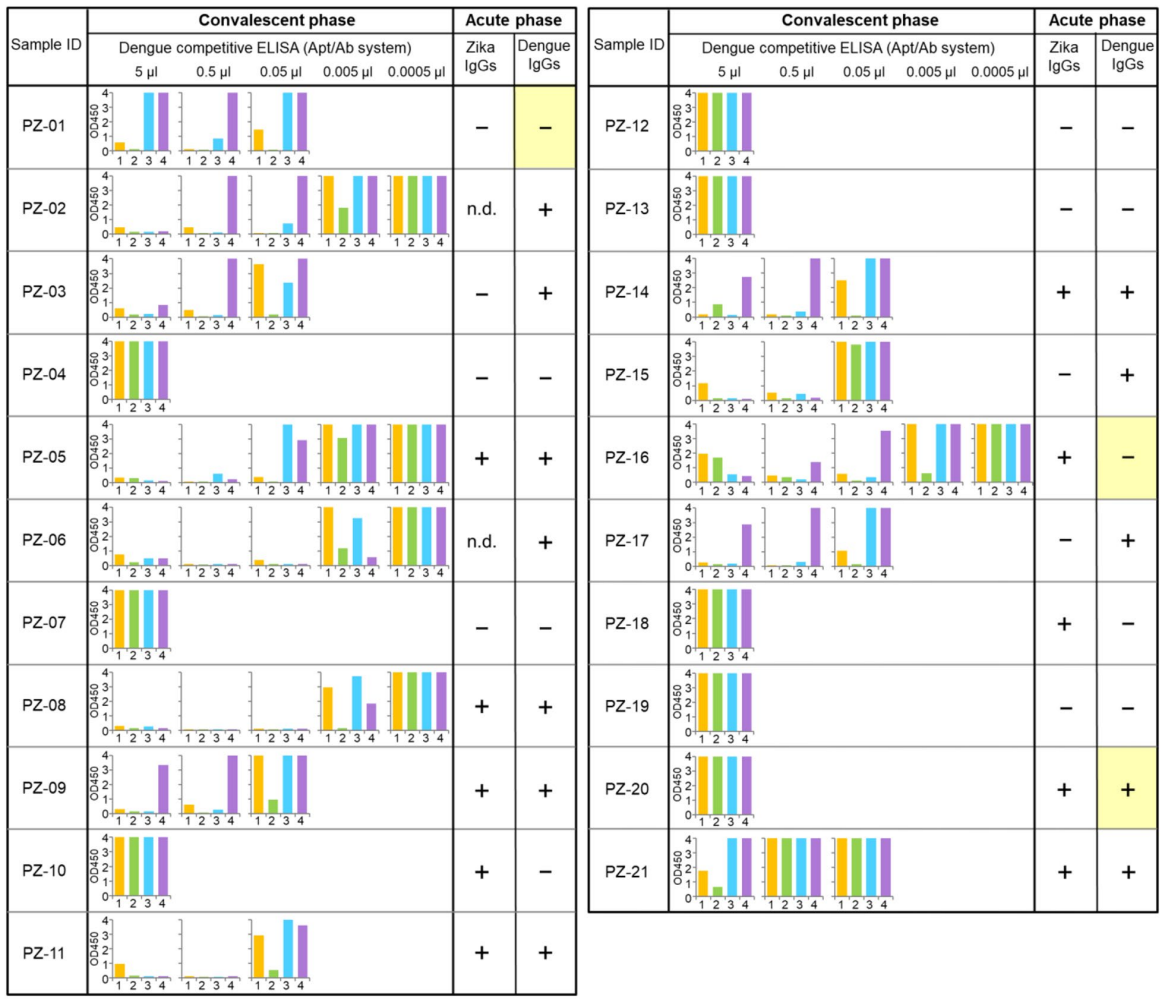
Serotype-specific anti-DENV-NS1 IgG detection using convalescent-phase ZIKV clinical samples by the competitive Apt/Ab ELISA format. For the competitive ELISA tests, 5 µL of diluted plasma samples (corresponding to the use of 0.0005–5 µL) were used and the amounts of the spiked DEN-NS1 were adjusted to obtain similar signal intensities, as shown in Supplementary Fig. 1. ZIKV IgG detection patterns in each acute-phase sample are from the ELISA results reported previously^74^ and Dengue IgG detection patterns are from the tests using the SD BIOLINE Dengue Duo kit (Dengue IgG/IgM test). *n.d.* not determined (not tested.).

Twenty-one plasma samples from ZIKV patients in the convalescent phase (10–14 days after the onset of symptoms [DPO]) in Singapore were examined with our dengue competitive ELISA (Supplementary Table 3 and Fig. 8). Since the acute-phase samples (2–7 DPO) corresponding to these patients were not available for this experiment, we used the previously obtained IgG detection data against Zika or dengue NS1 proteins in the acute phase (Supplementary Table 3 and Fig. 8). The anti-ZIKV-NS1 IgGs were detected by the H-zMut2 ELISA format^74^, which confirmed the positive results with anti-ZIKV-NS1 IgGs in all 21 convalescent-phase samples (Supplementary Table 3). The DEN-NS1 and IgM/IgGs to DENV were also tested by the SD BIOLINE Dengue Duo rapid IgG/IgM test (Dengue IgGs, Acute phase, Fig. 8 and Supplementary Table 3). However, the SD BIOLINE test uses mouse monoclonal anti-human IgG & IgM as the capture reagent on the membrane and gold colloid coated with the dengue virus envelope protein as the detector reagent, which could cross-react with IgGs to ZIKV^69,75,76^. Nineteen samples in the acute phase were negative for DEN-NS1 with the SD BIOLINE kit, and the other two samples, PZ-01 and PZ-05, were not tested.

Eight of the 21 samples (5 μL) from ZIKV patients did not inhibit the aptamer–DEN-NS1 binding for all dengue serotypes, and no cross-reactivity with the dengue competitive ELISA test was observed (Fig. 8). In contrast, the other 13 samples were positive in our competitive ELISA format with unique dengue serotype specificities, mostly serotype 2. Even small amounts (0.005–0.05 μL) of samples strongly inhibited the aptamer binding to DEN-NS1. In Singapore, many people could have been previously infected with DENV, especially serotype 2. Thus, these 13 patients may have had a prior DENV infection. The clinical samples from patients with the primary DENV infection and the secondary ZIKV infection reportedly showed strong cross-reactivity between ZIKA and DENV in the IgG detection^71^. Among these 13 samples, eleven acute phase samples were positive for the detection of the IgGs to DENV envelope proteins, using the SD BIOLINE test. Furthermore, three samples, PZ-03, PZ-15, and PZ-17, were negative for anti-ZIKV-NS1 IgG detection. Two samples, PZ-01 and PZ-16, showed a discrepancy between our competitive ELISA (anti-DEN-NS1 IgG positive in the convalescent phase) and the SD BIOLINE anti-DENV IgG test (negative to IgGs against DENV envelope proteins in the acute phase). PZ-20 revealed another discrepancy between our competitive ELISA (anti-DEN-NS1 IgG negative in the convalescent phase) and the SD BIOLINE test (positive to IgGs against DENV envelope proteins in the acute phase). These differences might result from the low sensitivity of the commercially available paper-based assay formats and the differences in the targeting DENV antigens. Together, these results suggest that our dengue competitive ELISA is not cross-reactive with anti-ZIKV-NS1 IgGs and can detect prior DENV infections in patients currently infected by ZIKV. We will further validate our method by comparisons with conventional methods and kits, using well-characterized clinical samples with sufficient patient information.

To confirm the absence of cross-reactivity of the competitive ELISA with anti-ZIKV-NS1 antibodies, we examined the inhibition by monoclonal antibodies, Ab#Z11 and Ab#Z12, prepared in-house for an antibody–antibody sandwich ELISA format to selectively detect ZIKV NS1 proteins. Both antibodies (100 fmol) showed no inhibition (Supplementary Fig. 7, top and middle panels). In contrast, the monoclonal antibody, Ab#D25 (Fig. 7a, used as the capture agent for the antibody–antibody sandwich ELISA system), effectively inhibited the aptamer–DEN-NS1 binding (Supplementary Fig. 7, bottom panel).

We also examined the cross-reactivity of our competitive ELISA with antibodies resulting from COVID-19 vaccination. During this research, the two healthy volunteers (PD0-1 and PD0-2), whose serums were used as controls (Fig. 3e,f), were vaccinated with the Pfizer-BioNTech COVID-19 vaccine. The production of SARS-CoV2 neutralizing antibodies was detected with a GenScript cPass kit (Supplementary Figs. 8a and b). Using these serum samples before and after vaccination, we tested our dengue competitive ELISA format and confirmed the absence of cross-reactivity with the COVID-19 vaccination in the competitive ELISA format (Supplementary Fig. 8c).

## Discussion

We have presented serotype-specific detection methods as a serologic test for IgG antibodies to DEN-NS1 in human blood samples, by the competitive ELISA format using high-affinity UB-DNA aptamers. Only IgGs with strong affinity to each of DEN-NS1 serotypes inhibit the DEN-NS1 detection by the UB-DNA aptamer‒antibody sandwich system, allowing for the serotype-specific IgG detection. This IgG detection provides valuable information for the dengue diagnostics and the development and use of the dengue vaccine. (1) The competitive ELISA format can identify the secondary infection within several days (during the febrile period) after fever onset. If anti-DEN-NS1 IgG antibodies are detected in patients within one week after fever onset, then this indicates a secondary infection that may warrant close monitoring. (2) The format can identify the serotypes in the late stage of the primary DENV infection and in the early stage of the secondary infection, in which the serotypes of the past infection can be determined. In combination with the antigen-detection ELISA format using the UB-DNA aptamer–antibody sandwich system^49^, the serotypes of both the current and past infections, as well as the infection status, could be determined in the early stage of DENV infection, enabling prompt and specific medical treatment. (3) The format can check whether a person has been previously infected with DENV and determine the serotype. In the currently used field test for dengue infection, the serotype identification is still optional. However, as shown by the increase in severity by secondary infection, rapid test kits that identify the serotype could facilitate the advancement of dengue epidemiology and pathology, by accumulating the data of the relationships among the serotypes, antibody production, and symptoms of dengue patients. (4) The format could also identify the dengue infection history of ZIKV-infected patients. Our data about the cross-reactivity with ZIKV infection using convalescent-phase clinical samples (Fig. 8) revealed two distinct patterns: one showed no cross-reactivity between dengue and Zika, in which patients were infected by only ZIKV, and the other exhibited strong inhibition in our format, in which patients might have been infected with DENV previously. For further evaluation, we will examine our dengue format using both the acute- and convalescent-phase ZIKV patient samples. (5) The format could provide valuable information for the usage and analyses of the dengue vaccines, for which documentation of prior infection is important prior to administration, due to concerns about antibody-dependent enhancement (ADE). For example, Dengvaxia can only be used in people previously infected with DENV, and thus the competitive ELISA can identify previous DENV infections. (6) The format could be useful for vaccine development to identify the specificity to antigen variants. To this end, the UB-DNA aptamer generation targeting viral particle or envelope proteins is more helpful.

Our tests using patient samples with secondary DENV infections (Fig. 4) revealed that the IgG antibodies responding to the past infection were produced predominantly, even upon secondary infections with different dengue serotypes. These results correlate with other reports^21–24,26,27,61^, supporting ADE where secondary heterologous infections occasionally result in severe symptoms and highlighting the risk of vaccinating dengue-naïve individuals. Patients with a primary infection produced IgG antibodies that mainly targeted the infected serotype. In the secondary infection, the initially produced IgG antibodies reacted more to the NS1 serotype of the past infection, and did not effectively react with the targets of the secondary infection. The application of this test in a larger cohort of dengue patients will clarify the mechanism of dengue pathogenesis, through the serotype-specific sequence of the DENV infection. Interestingly, this observation of patients with a secondary dengue infection is very similar to the observations of ZIKV patients who might have been previously infected by dengue (Fig. 8). In the future, our method may be expanded to test the efficacy of vaccine development^50,51^, and to diagnose other diseases and allergies.

This competitive ELISA format is a simple method using UB-DNA aptamers, which enable the quantitatively identification of serotype-specific IgGs. A similar IgG detection concept using conventional DNA aptamers was used to detect the IgG antibodies to the P48 protein of *Mycoplasma bovis*^77^. However, the affinities of the DNA aptamers to the target were relatively low (*K*_D_ = 16‒33 nM) due to the conventional 4-letter DNA aptamers, and thus the background in the IgG detection was high and the quantitative analysis was difficult. Furthermore, this competitive method using antibodies is also applicable to DNA aptamer generation, especially for cell-SELEX to selectively isolate aptamer candidates for desired targets on the cell-surface^78,79^.

For the serotype-specific detection by the competitive ELISA format, unbiased affinities and sensitivities of ligands to each serotype of DEN-NS1 proteins might be important. In the ExSELEX procedure, we generated four UB-DNA aptamers targeting each DEN-NS1 serotype by isolating each aptamer using the same selection pressure, in the combination of the ELISA-type sandwich system with the antibody (Ab#D06) pair^49^. Therefore, the ELISA formats for each DEN-NS1 serotype exhibit similar sensitivities: the limit of detection (LOD) values in 10% human serum is 6.97 ng/ml for DEN1-NS1, 6.91 ng/ml for DEN2-NS1, 11.06 ng/ml for DEN3-NS1, and 4.10 ng/ml for DEN4-NS1^49^.

Although the antibody–antibody sandwich system can also be used for the IgG detection, the sensitivity is lower than that of the UB-DNA aptamer–antibody sandwich system, especially in the early stage of infection, due to the biased affinities to each serotype (Fig. 7). In addition, this sensitivity difference between these systems might also result from the different binding modes between aptamer–target and antibody–target. In general, nucleic acid aptamers cover wider areas of target proteins, as compared to those covered by antibodies, and thus a series of IgGs that bind to a region within the wide area of the targets can inhibit the aptamer binding. Our competitive ELISA format using four serotype-specific UB-DNA aptamers can be used for the serotype-specific identification of IgGs with the lateral-flow assay (LFA) system, in which each aptamer is embedded in each slit on the single membrane as the capture agent.

As shown in Fig. 3, the commercially available human serum contains considerable amounts of anti-DEN-NS1 IgGs, presuming the widespread DENV epidemic (or endemic) where the samples were collected. This also draws attention to the use of serum as a control in epidemic and pandemic disease studies, such as the current COVID-19 pandemic. In our study, we used serum sample 2 (Fig. 3e) as the DENV-related IgG-free control serum.

The combination of this competitive ELISA for IgG detection with the original ELISA format for the serotype- or sub-serotype-specific DEN-NS1 detection^49^ allows the diagnosis of both past and current viral infections and will facilitate early medical care. Our competitive ELISA method could be employed for a wide range of IgG detections for infectious diseases, allergenic reactions and sensitivities, and vaccine development. By generating UB-DNA aptamers targeting each antigen or its variants, the competitive ELISA format using each aptamer/target pair could identify the specificity of IgGs produced by responses to infection, allergens, and vaccine treatments. Recently, SARS-CoV-2 serological assays with competitive or blocking ELISA formats, using the interaction between ACE2 and the viral spike protein, have been utilized to detect neutralization antibodies^80,81^ and are commercially available as a test kit (cPass from GenScript). However, there is a possibility that the serologic test kits have the problem of cross-reactivity between dengue and COVID-19 for both types of patients^9,10^. As noted above, UB-DNA aptamers cover a large area of target proteins, and our competitive ELISA format could detect not only the neutralization antibodies but also other IgGs that competitively bind to different target areas.

## Methods

### Oligonucleotides, proteins, and clinical samples

Biotinylated UB-DNA aptamers (AptD1, AptD2, AptD3, and AptD4, Fig. 2b and Supplementary Table 1) were chemically synthesized with an H8 DNA/RNA Synthesizer (K&A Laborgerate) in-house by using natural base, Biotin-dT, and Ds and Pa phosphoramidites. The natural base and Biotin-dT phosphoramidites were purchased from Glen Research and LGC Link Technologies. The Ds and Pa phosphoramidites were prepared in-house, as described previously^52,53^. The synthesized UB-DNA aptamers were purified by denaturing gel electrophoresis after deprotection. Recombinant DENV1–4 NS1 proteins (DEN1-NS1, DEN2-NS1, DEN3-NS1, and DEN4-NS1) were purchased from The Native Antigen Company. Control human serum was purchased from Sigma-Aldrich (Sigma #H4522, pooled) and obtained from a healthy Singaporean volunteer (human serum sample 1), or two different Japanese volunteers (human serum samples 2 and 3, corresponding to PD0-1 and PD0-2) who have never been infected by dengue viruses. Clinical samples (serum or plasma) used in this study were prepared from the whole-blood samples of the patients who consented to the study and provided written informed consent, referred by the Communicable Disease Center, TTSH. The study protocol was approved by the National Healthcare Group Domain Specific Review Board (references 2015/00528 and 2016/00076) and by the SingHealth Centralized Institutional Review Board (reference no. 2016/2219). Anti-dengue NS1 rabbit monoclonal antibodies, Ab#D06 and Ab#D25, and anti-Zika NS1 rabbit monoclonal antibodies, Ab#Z11 and Ab#Z12, were prepared in-house^49^. The Streptavidin-HRP conjugate (1 mg/mL) and Peroxidase-conjugated AffiniPure Goat Anti-Human IgG (H + L) (#109-035-003, 1 mg/mL) were obtained from Jackson ImmunoResearch. Streptavidin, Tween 20, BSA, and anti-rabbit IgG HRP conjugate (1 mg/mL) were obtained from Promega. The 10 × D-PBS solution was purchased from Nacalai Tesque.

The present experiments were approved by the institutions of A*STAR and NCID, and we confirm that all methods were performed in accordance with the guidelines and regulations of A*STAR and NCID/TTSH.

### ELISA using aptamer–antibody pair (Apt/Ab ELISA) for DEN-NS1 detection

All the incubation processes were performed at room temperature. Microtiter plates (Maxisorp 96-well plates from Thermo Fisher Scientific, #442404) were coated overnight with 100 µL/well of 10 µg/mL streptavidin in 0.1 M sodium carbonate buffer (pH 9.6). The streptavidin-coated wells were blocked with 300 µL of 10 mg/mL BSA in 1 × D-PBS for two hours, and then washed three times with 300 µL of washing buffer (20 mM Tris–HCl pH 7.5, 150 mM NaCl, 1 mM MgCl_2_, 2.7 mM KCl, 0.05% Tween 20). Each UB-DNA aptamer in dilution buffer (washing buffer supplemented with 1 mg/mL BSA) was immobilized on the streptavidin-coated wells by a 2-h incubation (100 µL, 15 nM AptD1 or 5 nM AptD2, AptD3, or AptD4), the wells were washed three times, each with 300 µL of washing buffer. To the aptamer-coated wells, 100 µL of an NS1–Ab#D06 mixture solution was added and incubated for 30 min. The mixture solutions were prepared beforehand, by a 30-min incubation of each NS1 protein in dilution buffer or human serum with 11.1 nM of Ab#D06 in dilution buffer supplemented with 2% Tween 20 (final concentration, dilution buffer 2) at 1: 9 ratios (vol/vol). After washing the wells once, 100 µL of secondary detector solution (anti-rabbit IgG HRP conjugate, diluted to 1: 2,500 with dilution buffer) was added to each well, and then incubated for 30 min. After washing the wells six times, each with 300 µL of washing buffer, 100 µL/well of SureBlue Reserve TMB Microwell Peroxidase Substrate (KPL) was added and incubated for 30 min. After adding 100 µL of 1 N HCl to each well to stop the reaction, the absorbance of the wells at 450 nm (OD_450_) was measured with a microplate reader, Cytation 3 (BioTek). The assays under each condition were performed in duplicate (n = 2), and the average absorbance data are shown in the graphs with error bars, which represent one standard deviation. When at least one of the two sample wells showed overflow (OD_450_ > 4.000), the data are shown with wavy lines in the graphs.

### Treatment of control human serum with protein A resin

The IgG in human serum was removed with protein A resin. Human serum from Sigma (500 μL, Lot#SLBT0310) was incubated with Amintra Protein A Resin (Expedeon, 500 µL of the slurry was washed three times, each with 1 mL of washing buffer) at room temperature for two hours with rotation. After the incubation, the resin was removed by centrifugation, and the supernatant was recovered and kept at 4 °C until use.

### Serology testing and DEN-NS1 detection using commercially available kits

As the controls, anti-dengue IgG and IgM serology detection and dengue NS1 detection were performed using commercially available lateral flow assays, Panbio Dengue Duo Cassette (Alere) [for DENV clinical samples], SD BIOLINE Dengue IgG/IgM rapid test (Abbott) [for ZIKV clinical samples], and SD BIOLINE Dengue NS1 Ag rapid test (Alere), respectively. For high-titer IgG and IgM detection (PanBio), 10 µL of each sample (human serum) was applied to the sample well of a Panbio Dengue Duo Cassette, and then two drops of the buffer included in the kit were immediately added. After 15 min, the test lines for IgG and IgM, as well as the control line, were checked visually, with the naked eye. For NS1 detection, 100 µL of each sample (human serum or human plasma) was applied to the sample well of SD BIOLINE Dengue NS1 Ag rapid test. After 20 min, the test and control lines were checked visually with the naked eye.

### Competitive IgG detection using Apt/Ab and Ab/Ab ELISA formats

For the assays by the Apt/Ab ELISA format, we used the wells coated with each UB-DNA aptamer as a capture agent. For the preparation of the loading samples, a serum sample (5 μL, directly or 10, 25, 50 or 100-fold diluted with dilution buffer) was first mixed with 0.5 μL of each NS1 protein (DEN1-NS1: 350 or 200 pg, DEN2-NS1: 350 or 500 pg, DEN3-NS1: 450 or 875 pg, DEN4-NS1 200 or 275 pg). The spiked NS1 protein amounts were adjusted to each DEN-NS1 protein lot used in the assays, so that the OD_450_ values only overflowed in dilution buffer, since the detection sensitivity of the spiked NS1 varied depending on the purchased DEN-NS1’s lot (Supplementary Fig. 1). After a 30-min incubation at 25 °C, the solution was mixed with 45 µL of 11.1 nM Ab#D06 in dilution buffer 2 and incubated for 30 min, followed by loading into the aptamer-coated well (50 µL) and a 30-min incubation. The subsequent procedures were performed as described above for the Apt/Ab ELISA.

For the assays by the Ab/Ab ELISA format, we used the wells coated with Ab#D25 (overnight) as a capture agent. For the preparation of the loading samples, a serum sample (5 μL, directly or 10, 25, 50 or 100-fold diluted with dilution buffer) was first mixed with 0.5 μL of each NS1 protein (DEN1-NS1: 400 pg, DEN2-NS1: 250 pg, DEN3-NS1: 400 pg, DEN4-NS1: 300 pg). The spiked NS1 protein amounts were adjusted by each DEN-NS1 protein so that the OD_450_ values only overflowed in the 10% control human serum sample, since the background signal levels were different between dilution buffer and 10% human serum (Supplementary Fig. 1)^49^. After a 30-min incubation at 25 °C, 45 µL of the solution was mixed with 11.1 nM biotinylated Ab#D06 in dilution buffer 2. The subsequent procedures were performed as described above for the Ab/Ab ELISA.

From the plots of OD_450_ against the volume of human serum used in the ELISA, the relative IgG activity was calculated through normalization of the serum volume required to exhibit an OD_450_ lower than 1.0 (5/[the serum volume required for an OD_450_ of 1.0]), as shown in Supplementary Fig. 2.

### Typical conventional method for IgG detection by ELISA

For the assays, we prepared the wells coated with each DEN-NS1, by a 2-h incubation of 1 µg/mL DEN-NS1 (The Native Antigen Company, 50 µL/well) in 0.1 M sodium carbonate buffer (pH 9.6) at 25 °C, followed by blocking with BSA. A serum sample (0.05 to 5 μL) in dilution buffer (50 µL) was loaded in the well and incubated for 30 min at 25 °C. After washing the wells once, 100 µL of secondary detector solution (Peroxidase-conjugated AffiniPure Goat Anti-Human IgG (H + L), diluted 1: 2,500 with dilution buffer) was added to each well and then incubated for 30 min, followed by TMB detection as described above in the Apt/Ab and Ab/Ab ELISA formats.

## Supplementary Information

Supplementary Information.

## References

[1] Maple PAC, Sikora K (2021). How useful is COVID-19 antibody testing—A current assessment for oncologists. Clin. Oncol..

[2] Balestri R, Magnano M, Rizzoli L, Rech G (2020). Do we have serological evidences that chilblain-like lesions are related to SARS-CoV-2? A review of the literature. Dermatol Ther.

[3] Deeks JJ (2020). Antibody tests for identification of current and past infection with SARS-CoV-2. Cochrane Database Syst. Rev..

[4] Espejo AP (2020). Review of current advances in serologic testing for COVID-19. Am. J. Clin. Pathol..

[5] Motley MP, Bennett-Guerrero E, Fries BC, Spitzer ED (2020). Review of viral testing (polymerase chain reaction) and antibody/serology testing for severe acute respiratory syndrome-coronavirus-2 for the intensivist. Crit. Care Explor..

[6] Goldberg ME, Djavadi-Ohaniance L (1993). Methods for measurement of antibody/antigen affinity based on ELISA and RIA. Curr. Opin. Immunol..

[7] Kozel TR, Burnham-Marusich AR (2017). Point-of-care testing for infectious diseases: Past, present, and future. J. Clin. Microbiol..

[8] Pang J, Chia PY, Lye DC, Leo YS (2017). Progress and challenges towards point-of-care diagnostic development for dengue. J. Clin. Microbiol..

[9] Masyeni S (2021). Serological cross-reaction and coinfection of dengue and COVID-19 in Asia: Experience from Indonesia. Int. J. Infect. Dis..

[10] Yan G (2020). Covert COVID-19 and false-positive dengue serology in Singapore. Lancet Infect. Dis..

[11] Bhatt S (2013). The global distribution and burden of dengue. Nature.

[12] Ang LW (2019). A 15-year review of dengue hospitalizations in Singapore: Reducing admissions without adverse consequences, 2003 to 2017. PLoS Negl. Trop. Dis..

[13] Halstead SB, O'Rourke EJ (1977). Dengue viruses and mononuclear phagocytes. I. Infection enhancement by non-neutralizing antibody. J. Exp. Med..

[14] Halstead SB, O'Rourke EJ (1977). Antibody-enhanced dengue virus infection in primate leukocytes. Nature.

[15] Halstead SB (1979). In vivo enhancement of dengue virus infection in rhesus monkeys by passively transferred antibody. J. Infect. Dis..

[16] Halstead SB (2007). Dengue. Lancet.

[17] Guzman MG, Harris E (2015). Dengue. Lancet.

[18] Guzman MG, Gubler DJ, Izquierdo A, Martinez E, Halstead SB (2016). Dengue infection. Nat. Rev. Dis. Primers.

[19] Wilder-Smith A, Ooi EE, Horstick O, Wills B (2019). Dengue. Lancet.

[20] Priyamvada L (2016). Human antibody responses after dengue virus infection are highly cross-reactive to Zika virus. Proc. Natl. Acad. Sci. U S A.

[21] Mathew A (2011). B-cell responses during primary and secondary dengue virus infections in humans. J. Infect. Dis..

[22] Corbett KS, Katzelnick L, Tissera H, Amerasinghe A, de Silva AD, de Silva AM (2015). Preexisting neutralizing antibody responses distinguish clinically inapparent and apparent dengue virus infections in a Sri Lankan pediatric cohort. J. Infect. Dis..

[23] Priyamvada L (2016). B cell responses during secondary dengue virus infection are dominated by highly cross-reactive, memory-derived plasmablasts. J. Virol..

[24] Katzelnick LC (2017). Antibody-dependent enhancement of severe dengue disease in humans. Science.

[25] St. John AL, Rathore APS (2019). Adaptive immune responses to primary and secondary dengue virus infections. Nat. Rev. Immunol..

[26] Patel B, Longo P, Miley MJ, Montoya M, Harris E, de Silva AM (2017). Dissecting the human serum antibody response to secondary dengue virus infections. PLoS Negl. Trop. Dis..

[27] Reich NG (2013). Interactions between serotypes of dengue highlight epidemiological impact of cross-immunity. J. R. Soc. Interface.

[28] Villar L (2015). Efficacy of a tetravalent dengue vaccine in children in Latin America. N. Engl. J. Med..

[29] Capeding MR (2014). Clinical efficacy and safety of a novel tetravalent dengue vaccine in healthy children in Asia: a phase 3, randomised, observer-masked, placebo-controlled trial. Lancet.

[30] Ferguson NM, Rodriguez-Barraquer I, Dorigatti I, Mier YT-RL, Laydon DJ, Cummings DA (2016). Benefits and risks of the Sanofi-Pasteur dengue vaccine: Modeling optimal deployment. Science.

[31] Sridhar S (2018). Effect of dengue serostatus on dengue vaccine safety and efficacy. N. Engl. J. Med..

[32] Aguiar M, Halstead SB, Stollenwerk N (2017). Consider stopping dengvaxia administration without immunological screening. Exp. Rev. Vaccines.

[33] Halstead SB (2017). Dengvaxia sensitizes seronegatives to vaccine enhanced disease regardless of age. Vaccine.

[34] Luo R, Fongwen N, Kelly-Cirino C, Harris E, Wilder-Smith A, Peeling RW (2019). Rapid diagnostic tests for determining dengue serostatus: A systematic review and key informant interviews. Clin. Microbiol. Infect..

[35] Muller DA, Depelsenaire AC, Young PR (2017). Clinical and laboratory diagnosis of dengue virus infection. J. Infect. Dis..

[36] Peeling RW (2010). Evaluation of diagnostic tests: Dengue. Nat. Rev. Microbiol..

[37] Lebani K (2017). Isolation of serotype-specific antibodies against dengue virus non-structural protein 1 using phage display and application in a multiplexed serotyping assay. PLoS ONE.

[38] Roltgen K (2018). Development of dengue virus serotype-specific NS1 capture assays for the rapid and highly sensitive identification of the infecting serotype in human sera. J. Immunol..

[39] Bosch I (2020). Serotype-specific detection of dengue viruses in a nonstructural protein 1-based enzyme-linked immunosorbent assay validated with a multi-national cohort. PLoS Negl. Trop. Dis..

[40] Bosch I (2017). Rapid antigen tests for dengue virus serotypes and Zika virus in patient serum. Sci. Transl. Med..

[41] Ng DHL (2020). Fever patterns, cytokine profiles, and outcomes in COVID-19. Open Forum Infect. Dis..

[42] Stringari LL (2021). Covert cases of severe acute respiratory syndrome coronavirus 2: An obscure but present danger in regions endemic for dengue and chikungunya viruses. PLoS ONE.

[43] Ellington AD, Szostak JW (1990). *In vitro* selection of RNA molecules that bind specific ligands. Nature.

[44] Tuerk C, Gold L (1990). Systematic evolution of ligands by exponential enrichment: RNA ligands to bacteriophage T4 DNA polymerase. Science.

[45] Kimoto M, Yamashige R, Matsunaga K, Yokoyama S, Hirao I (2013). Generation of high-affinity DNA aptamers using an expanded genetic alphabet. Nat. Biotechnol..

[46] Matsunaga K, Kimoto M, Hirao I (2017). High-affinity DNA aptamer generation targeting von Willebrand factor A1-domain by genetic alphabet expansion for systematic evolution of ligands by exponential enrichment using two types of libraries composed of five different bases. J. Am. Chem. Soc..

[47] Hirao I, Kimoto M, Lee KH (2018). DNA aptamer generation by ExSELEX using genetic alphabet expansion with a mini-hairpin DNA stabilization method. Biochimie.

[48] Matsunaga K, Kimoto M, Hanson C, Sanford M, Young HA, Hirao I (2015). Architecture of high-affinity unnatural-base DNA aptamers toward pharmaceutical applications. Sci. Rep..

[49] Matsunaga K (2021). High-affinity five/six-letter DNA aptamers with superior specificity enabling the detection of dengue NS1 protein variants beyond the serotype identification. Nucleic Acids Res. (in press).

[50] Sharma M (2019). Magnitude and functionality of the NS1-specific antibody response elicited by a live-attenuated tetravalent dengue vaccine candidate. J. Infect. Dis..

[51] Halstead SB, Russell PK, Brandt WE (2019). NS1, dengue's dagger. J. Infect. Dis..

[52] Hirao I (2006). An unnatural hydrophobic base pair system: Site-specific incorporation of nucleotide analogs into DNA and RNA. Nat. Methods.

[53] Kimoto M, Soh SHG, Tan HP, Okamoto I, Hirao I (2021). Cognate base-pair selectivity of hydrophobic unnatural bases in DNA ligation by T4 DNA ligase. Biopolymers.

[54] Hirao I, Nishimura Y, Naraoka T, Watanabe K, Arata Y, Miura K (1989). Extraordinary stable structure of short single-stranded DNA fragments containing a specific base sequence: d(GCGAAAGC). Nucleic Acids Res..

[55] Hirao I (1994). Most compact hairpin-turn structure exerted by a short DNA fragment, d(GCGAAGC) in solution: An extraordinarily stable structure resistant to nucleases and heat. Nucleic Acids Res..

[56] Yoshizawa S, Kawai G, Watanabe K, Miura K, Hirao I (1997). GNA trinucleotide loop sequences producing extraordinarily stable DNA minihairpins. Biochemistry.

[57] Kimoto M, Nakamura M, Hirao I (2016). Post-ExSELEX stabilization of an unnatural-base DNA aptamer targeting VEGF165 toward pharmaceutical applications. Nucleic Acids Res..

[58] Hamashima K, Kimoto M, Hirao I (2018). Creation of unnatural base pairs for genetic alphabet expansion toward synthetic xenobiology. Curr. Opin. Chem. Biol..

[59] Kimoto M, Shermane Lim YW, Hirao I (2019). Molecular affinity rulers: Systematic evaluation of DNA aptamers for their applicabilities in ELISA. Nucleic Acids Res..

[60] Chao DY, Galula JU, Shen WF, Davis BS, Chang GJ (2015). Nonstructural protein 1-specific immunoglobulin M and G antibody capture enzyme-linked immunosorbent assays in diagnosis of flaviviral infections in humans. J. Clin. Microbiol..

[61] Shu PY (2003). Comparison of capture immunoglobulin M (IgM) and IgG enzyme-linked immunosorbent assay (ELISA) and nonstructural protein NS1 serotype-specific IgG ELISA for differentiation of primary and secondary dengue virus infections. Clin. Diagn. Lab. Immunol..

[62] Tyson J (2019). Combination of nonstructural protein 1-based enzyme-linked immunosorbent assays can detect and distinguish various dengue virus and zika virus infections. J. Clin. Microbiol..

[63] Raafat N, Blacksell SD, Maude RJ (2019). A review of dengue diagnostics and implications for surveillance and control. Trans. R. Soc. Trop. Med. Hyg..

[64] Blacksell SD (2012). Comparison of seven commercial antigen and antibody enzyme-linked immunosorbent assays for detection of acute dengue infection. Clin. Vaccine Immunol..

[65] Azimzadeh A, Weiss E, Van Regenmortel MH (1992). Measurement of affinity of viral monoclonal antibodies using Fab'-peroxidase conjugate. Influence of antibody concentration on apparent affinity. Mol Immunol.

[66] Zhang L, Li Z, Jin H, Hu X, Su J (2018). Development and application of a monoclonal antibody-based blocking ELISA for detection of antibodies to Tembusu virus in multiple poultry species. BMC Vet. Res..

[67] Chang SF (2008). Retrospective serological study on sequential dengue virus serotypes 1 to 4 epidemics in Tainan City, Taiwan, 1994 to 2000. J. Microbiol. Immunol. Infect..

[68] Chia PY (2017). Clinical features of patients with Zika and dengue virus co-infection in Singapore. J. Infect..

[69] Tsai WY (2017). Distinguishing secondary dengue virus infection from Zika virus infection with previous dengue by a combination of 3 simple serological tests. Clin. Infect. Dis..

[70] Gao X (2018). Delayed and highly specific antibody response to nonstructural protein 1 (NS1) revealed during natural human ZIKV infection by NS1-based capture ELISA. BMC Infect. Dis..

[71] Felix AC (2017). Cross reactivity of commercial anti-dengue immunoassays in patients with acute Zika virus infection. J. Med. Virol..

[72] Dejnirattisai W (2016). Dengue virus sero-cross-reactivity drives antibody-dependent enhancement of infection with zika virus. Nat. Immunol..

[73] Fernandez E (2017). Human antibodies to the dengue virus E-dimer epitope have therapeutic activity against Zika virus infection. Nat. Immunol..

[74] Yap TL (2021). Engineered NS1 for sensitive, specific zika virus diagnosis from patient serology. Emerg. Infect. Dis..

[75] Chao DY, Whitney MT, Davis BS, Medina FA, Munoz JL, Chang GJ (2019). Comprehensive evaluation of differential serodiagnosis between zika and dengue viral infections. J. Clin. Microbiol..

[76] Zaidi MB (2020). Serological tests reveal significant cross-reactive human antibody responses to Zika and Dengue viruses in the Mexican population. Acta Trop.

[77] Fu P (2014). Enzyme linked aptamer assay: Based on a competition format for sensitive detection of antibodies to *Mycoplasma bovis* in serum. Anal Chem.

[78] Zumrut HE, Ara MN, Maio GE, Van NA, Batool S, Mallikaratchy PR (2016). Ligand-guided selection of aptamers against T-cell receptor-cluster of differentiation 3 (TCR-CD3) expressed on Jurkat.E6 cells. Anal. Biochem..

[79] Zumrut HE, Mallikaratchy PR (2020). Ligand guided selection (LIGS) of artificial nucleic acid ligands against cell surface targets. ACS Appl. Bio Mater..

[80] Tan CW (2020). A SARS-CoV-2 surrogate virus neutralization test based on antibody-mediated blockage of ACE2-spike protein-protein interaction. Nat. Biotechnol..

[81] Byrnes, J.R*. et al.* A SARS-CoV-2 serological assay to determine the presence of blocking antibodies that compete for human ACE2 binding. *medRxiv* (2020) (**in press**).

