# Supplementary material for: Competitive ELISA for a serologic test to detect dengue serotype-specific anti-NS1 IgGs using high-affinity UB-DNA aptamers
Source: Sci Rep. 2021 Sep 9;11:18000. doi: 10.1038/s41598-021-97339-8 (PMC8429655; doi:10.1038/s41598-021-97339-8)
Supplement: Supplementary file 1 — Supplementary Information. [file 41598_2021_97339_MOESM1_ESM.pdf]

Supplementary Information

Competitive ELISA for a serological test to detect dengue serotype-specific anti-NS1 IgGs using high-affinity UB-DNA aptamers

Ken-ichiro Matsunaga,[1] Michiko Kimoto,[1] Vanessa Weixun Lim,[2] Tun-Linn Thein[2], Shawn Vasoo,[2,3,4] Yee-Sin Leo,[2,3,4,5] William Sun,[1,6] and Ichiro Hirao[1,*]

[1]Institute of Bioengineering and Bioimaging, 31 Biopolis Way, The Nanos, #07-01, Singapore 138669, Singapore

[2]National Centre for Infectious Diseases, 16 Jalan Tan Tock Seng, Singapore 308442, Singapore

[3]Department of Infectious Diseases, Tan Tock Seng Hospital, 11 Jalan Tan Tock Seng, Singapore 308433, Singapore

[4]Lee Kong Chian School of Medicine, Nanyang Technological University, 59 Nanyang Dr., Experimental Medicine Building, Singapore 636921, Singapore

[5]Saw Swee Hock School of Public Health, National University of Singapore, 12 Science Drive 2, #10-01, Singapore 117549, Singapore

[6]Department of Physiology, Yong Loo Lin School of Medicine, National University of Singapore, Singapore 117593, Singapore

* To whom correspondence should be addressed. Tel: +65-6824-7104; Fax: +65-6478-9083; Email: ichiro@ibb.a-star.edu.sg

Supplementary Table 1: Sequences of UB-DNA aptamers that bind to each DEN-NS1 serotype.

Supplementary Table 2: Patient sample information (DENV infection).

Supplementary Table 3: Patient sample information (ZIKV infection)

Supplementary Figure 1: Quantification of the high-affinity anti-DEN-NS1 IgG activities for the competitive ELISA.

Supplementary Figure 2: Determination of the amount of each DEN-NS1 spiked in the competitive ELISA.

Supplementary Figure 3: Competitive ELISA using Apt/Ab pair for the patient samples, PD1-2, PD1-3, and PD2-2.

Supplementary Figure 4: Competitive ELISA using Apt/Ab pair for the patient sample, PD2-3.

Supplementary Figure 5: Competitive ELISA using Apt/Ab pair for the patient sample, PD3-3.

Supplementary Figure 6: Competitive ELISA using Apt/Ab pair for the patient samples, PD3-1 and PD4-1.

Supplementary Figure 7: Competitive ELISA using Apt/Ab pair for the control samples spiked with monoclonal antibodies against ZIKV and DENV NS1 proteins.

Supplementary Figure 8: Competitive ELISA using Apt/Ab pair for the COVID-19 vaccinated samples.

Supplementary Table 1: Sequences of UB-DNA aptamers that bind to each DEN-NS1 serotype. The stem regions in the aptamers are highlighted in yellow, and the mini-hairpin DNA sequences are indicated in bold.

Name	Length	Sequence (5'- to -3': **L** = Biotin-dT, **x** = dDs, **y** = dPa)
AptD1	48-mer	CCCCAGACGGACTGGTGT**x**CTCGG**x**ATGGCCGTCTGGGG**CGCGLAGCG**
AptD2	72-mer	GGCTGGTCCG**x**CTGGGAACAAG**x**GGCGGGAGGGA**y**GGGTGTGGGTGCGACAAG CGGACCAGCC**CGCGLAGCG**
AptD3	59-mer	CCGCTTGTCATCTA**x**CCTGGCC**x**TGTGGTACTGTAACGGCTGACAAGCGG**CGC GLAGCG**
AptD4	57-mer	CGGCGGAGACGTAACGC**x**TATCAAATC**x**AAACAGCTTAGGGTCCGCCG**CGCGL AGCG**

Supplementary Table 2. Patient sample information (DENV infection). The recruited patients were tested and confirmed as Dengue NS1 positive by routine hospital diagnostics, using the SD BIOLINE NS1 Ag rapid test (Alere), and had fevers within 3–5 days from illness onset. The current infection by the dengue virus and the serotypes (Serotype) were reconfirmed by an RT-qPCR analysis and sequencing of the RT-PCR products. The high-titer IgG and IgM detection with the Panbio Dengue Duo Cassette (Panbio LFA) was tested to judge whether the current infection is presumed as primary (IgG negative) or secondary infection (IgG positive), as described in the Materials and Methods. The patient samples with asterisks, continuously collected after the illness onset, were used to investigate the IgG status with our competitive ELISA formats (see Figures 4 and 7, Supplementary Figures 3−6).

Sample		Fever day	Serotype	Panbio LFA		
				IgM	IgG	Primary or Secondary
PD1-1	Serum	5	D1	+	+	Secondary
PD1-2*	Serum	4	D1	+	-	Primary
PD1-3*	Serum	4	D1	+	-	Primary
PD1-4	Serum	5	D1	+	-	Primary
PD1-5	Serum	4	D1	-	-	Primary
PD1-6	Serum	3	D1	-	-	Primary
PD1-9	Serum	4	D1	+	+	Secondary
PD1-10	Serum	5	D1	+	-	Primary
PD1-11	Serum	5	D1	+	-	Primary
PD1-12	Serum	3	D1	+	-	Primary
PD1-14	Serum	4	D1	-	+	Secondary
PD1-15	Serum	3	D1	-	-	Primary
PD1-17	Serum	4	D1	-	-	Primary
PD1-18	Serum	5	D1	+	-	Primary
PD1-19	Serum	4	D1	+	+	Secondary
PD1-20	Serum	5	D1	+	-	Primary
PD1-22	Serum	3	D1	-	-	Primary
PD2-1	Plasma	3	D2	+	-	Primary
PD2-2*	Serum	3	D2	-	-	Primary
PD2-3*	Serum	5	D2	+	+	Secondary
PD2-4	Plasma	4	D2	-	-	Primary
PD3-1*	Serum	5	D3	+	-	Primary
PD3-2	Serum	4	D3	+	+	Secondary
PD3-3*	Serum	4	D3	-	-	Primary
PD3-4	Serum	5	D3	-	+	Secondary
PD4-1*	Serum	3	D4	+	+	Secondary

Supplementary Table 3. Patient sample information (ZIKV infection). The patients were confirmed to be infected with Zika virus (ZIKV) by an RT-qPCR analysis using their plasma and urine samples collected during their first visits (in the acute phase), and also tested by the SD BIOLINE Dengue Duo rapid test from Abbott (Dengue NS1 Ag test and Dengue IgG/IgM test). We used 42 samples from 21 unique patients with ZIKV, collected at two phases: acute (2–7 days post onset of symptoms [DPO]) and convalescent (11–14 DPO). The positive/negative ZIKV IgG result for each sample was obtained by H-zMut2 ELISA (Yap, T.L., et al. *Emerging Infectious Diseases*, 27, 2021, 1427–1437). Previous dengue infection of each patient was determined by our dengue competitive ELISA format (Ap/Ab system) using the convalescent-phase samples, as shown in Figure 8. NT: not tested.

Sample ID		Acute phase					Convalescent phase		
		DPO	H-zMut2 ELISA ZIKV IgG	DENV NS1	DENV IgG	DENV IgM	DPO	H-zMut2 ELISA ZIKV IgG	Presumed previous dengue infection
PZ-01	Plasma	5	-	NT	-	-	12	+	DENV2
PZ-02	Plasma	7	NT	-	+	+	14	+	DENV2
PZ-03	Plasma	3	-	-	+	+	12	+	DENV2
PZ-04	Plasma	5	-	-	-	+	14	+	No
PZ-05	Plasma	5	+	NT	+	+	12	+	DENV2
PZ-06	Plasma	7	NT	-	+	+	10	+	DENV2/4
PZ-07	Plasma	3	-	-	-	+	12	+	No
PZ-08	Plasma	3	+	-	+	-	12	+	DENV2
PZ-09	Plasma	5	+	-	+	-	13	+	DENV2
PZ-10	Plasma	4	+	-	-	-	12	+	No
PZ-11	Plasma	5	+	-	+	-	11	+	DENV2
PZ-12	Plasma	6	-	-	-	+	11	+	No
PZ-13	Plasma	6	-	-	-	-	12	+	No
PZ-14	Plasma	5	+	-	+	-	11	+	DENV2
PZ-15	Plasma	4	-	-	+	+	13	+	DENV1/2/3/4
PZ-16	Plasma	4	+	-	-	-	12	+	DENV2
PZ-17	Plasma	4	-	-	+	+	12	+	DENV2
PZ-18	Plasma	3	+	-	-	-	11	+	No
PZ-19	Plasma	2	-	-	-	-	12	+	No
PZ-20	Plasma	4	+	-	+	-	12	+	No
PZ-21	Plasma	7	+	-	+	-	13	+	DENV2

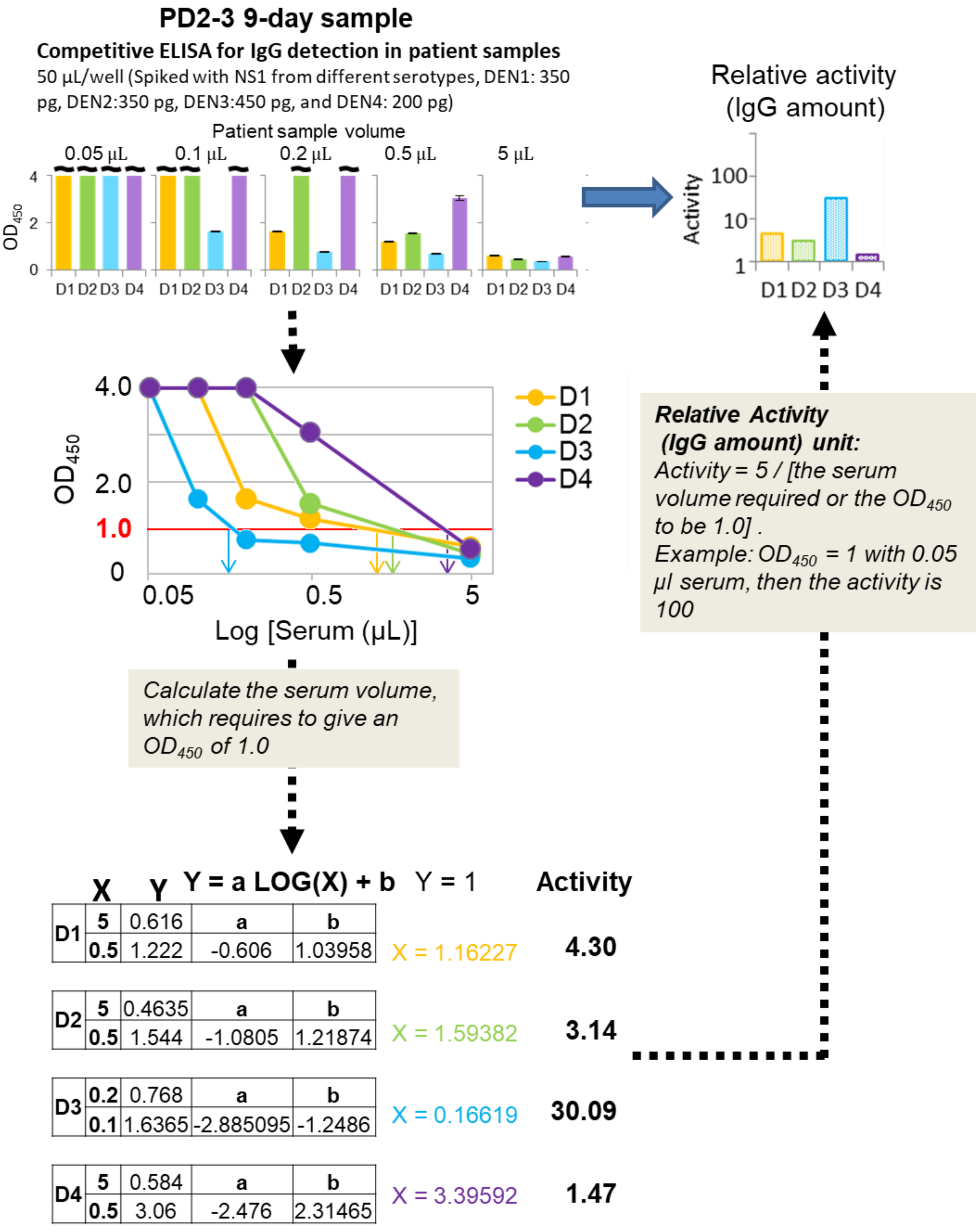

Supplementary Figure 1. Quantification of the high-affinity anti-DEN-NS1 IgG activities for the competitive ELISA. First, the serum or plasma samples (0.05 to 5 µL) are subjected to competitive-inhibition ELISA in the presence of a defined amount of each DEN-NS1 serotype (The Native Antigen Company). From the OD_{450} values against each sample volume, the required volume corresponding to an OD_{450} of 1.0 is determined. Then, the IgG relative activity is then calculated according to the formula: Activity = 5 / (the serum volume required for an OD_{450} of 1.0).

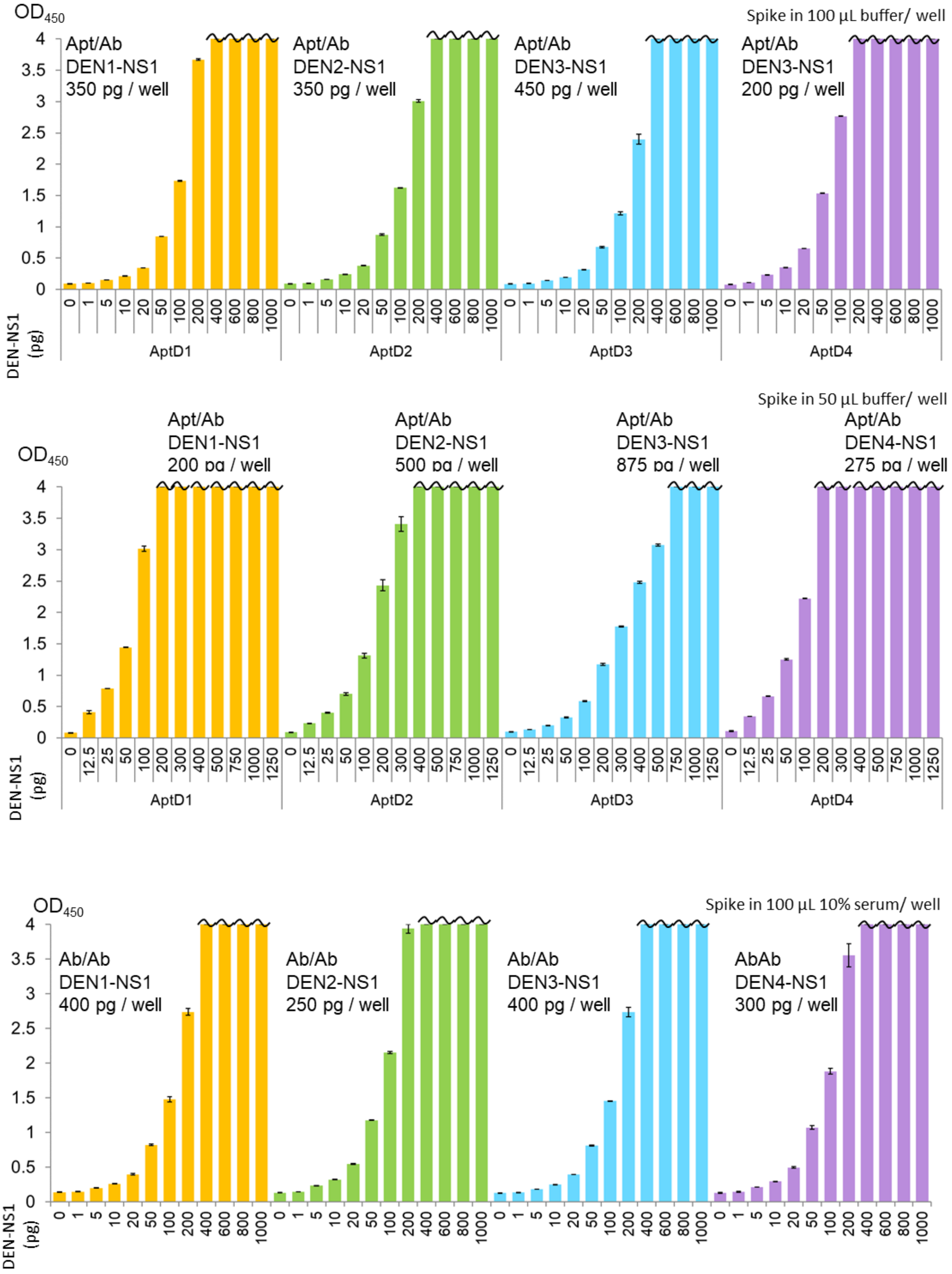

Supplementary Figure 2. Determination of the amount of each DEN-NS1 spiked in the competitive ELISA. The amount of each spiked DEN-NS1 was adjusted for the competitive ELISA format. For examples, the top-panel conditions were used for the Apt/Ab competitive ELISAs in Figures 3, 4, 5, and some of Figure 6 (shown in Figure 4). The middle-panel conditions were used for the Apt/Ab competitive ELISA for the other samples in Figure 6 (PD1-4, PD1-5, PD1-6, PD1-9, PD1-10, PD1-11, PD1-12, PD1-14, PD1-15, PD1-17, PD1-18, PD1-19, PD1-20, and PD1-22).

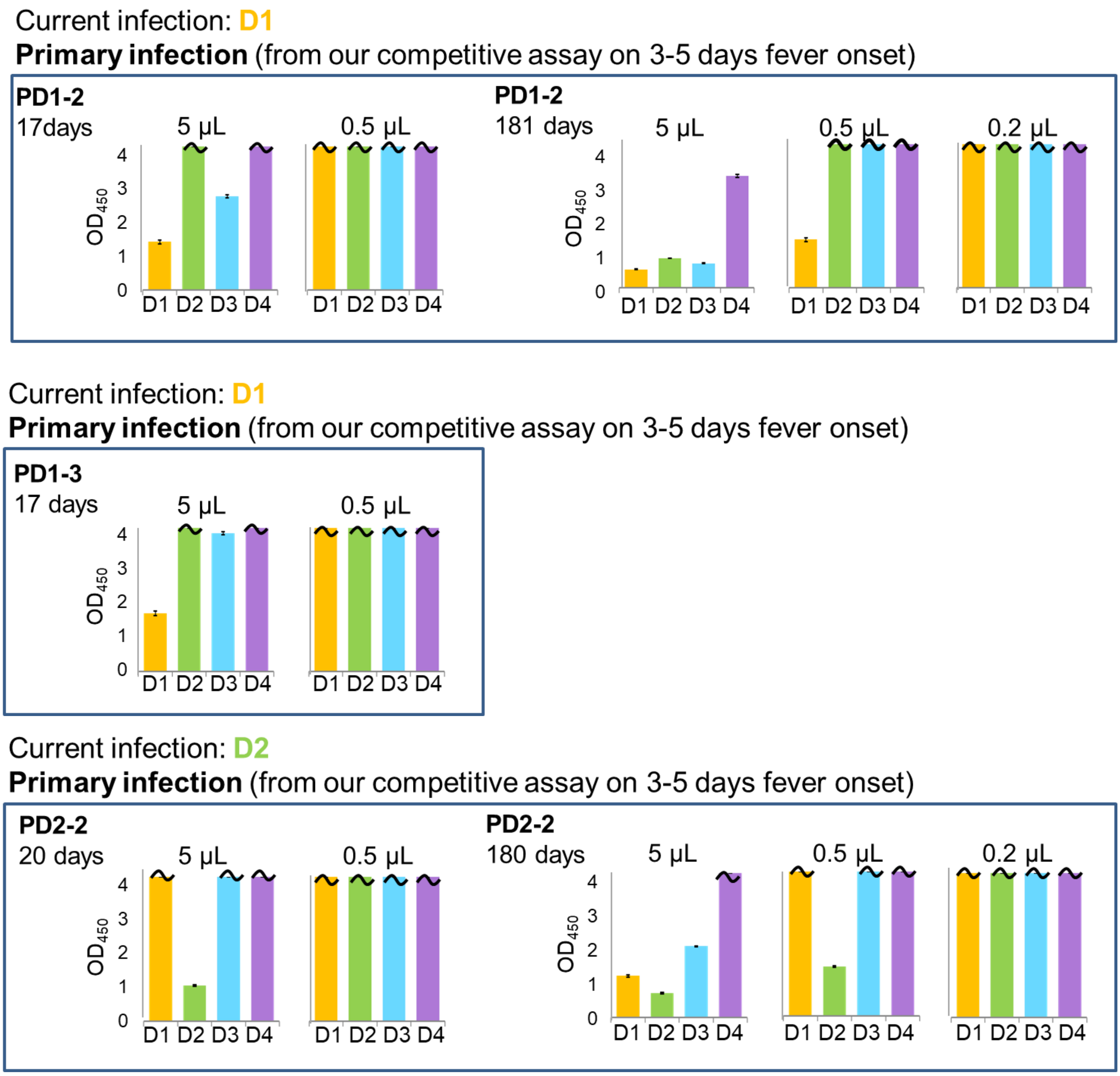

Supplementary Figure 3. Competitive ELISA using Apt/Ab pair for the patient samples, PD1-2, PD1-3, and PD2-2. Anti-DEN-NS1 IgG activities were quantified from these data and summarized in Figure 4.

Current infection: D2

Secondary infection (Past D3: from our competitive assay on 3-5 days fever onset)

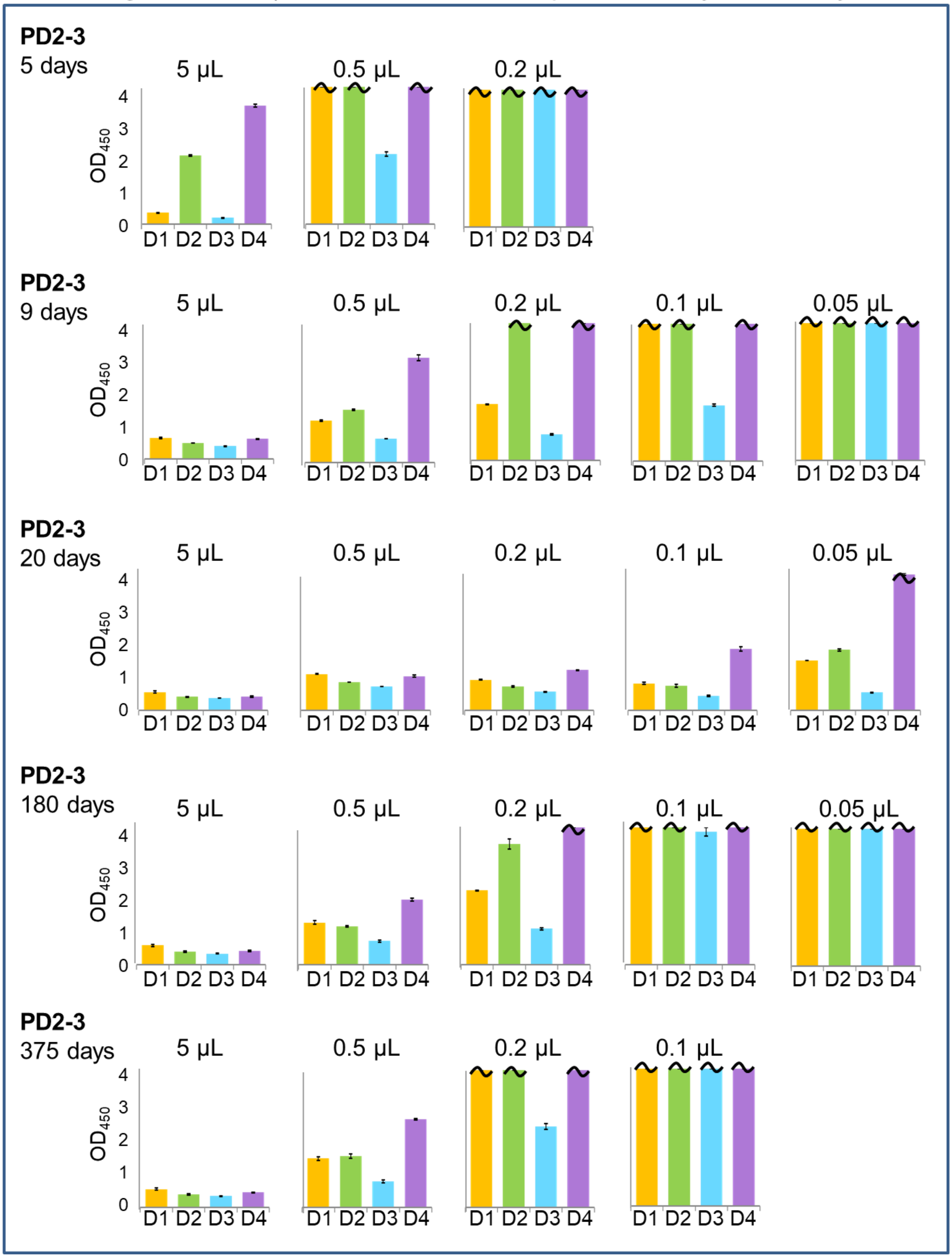

Supplementary Figure 4. Competitive ELISA using Apt/Ab pair for the patient sample, PD2-3. Anti-DEN-NS1 IgG activities were quantified from these data and summarized in Figure 4.

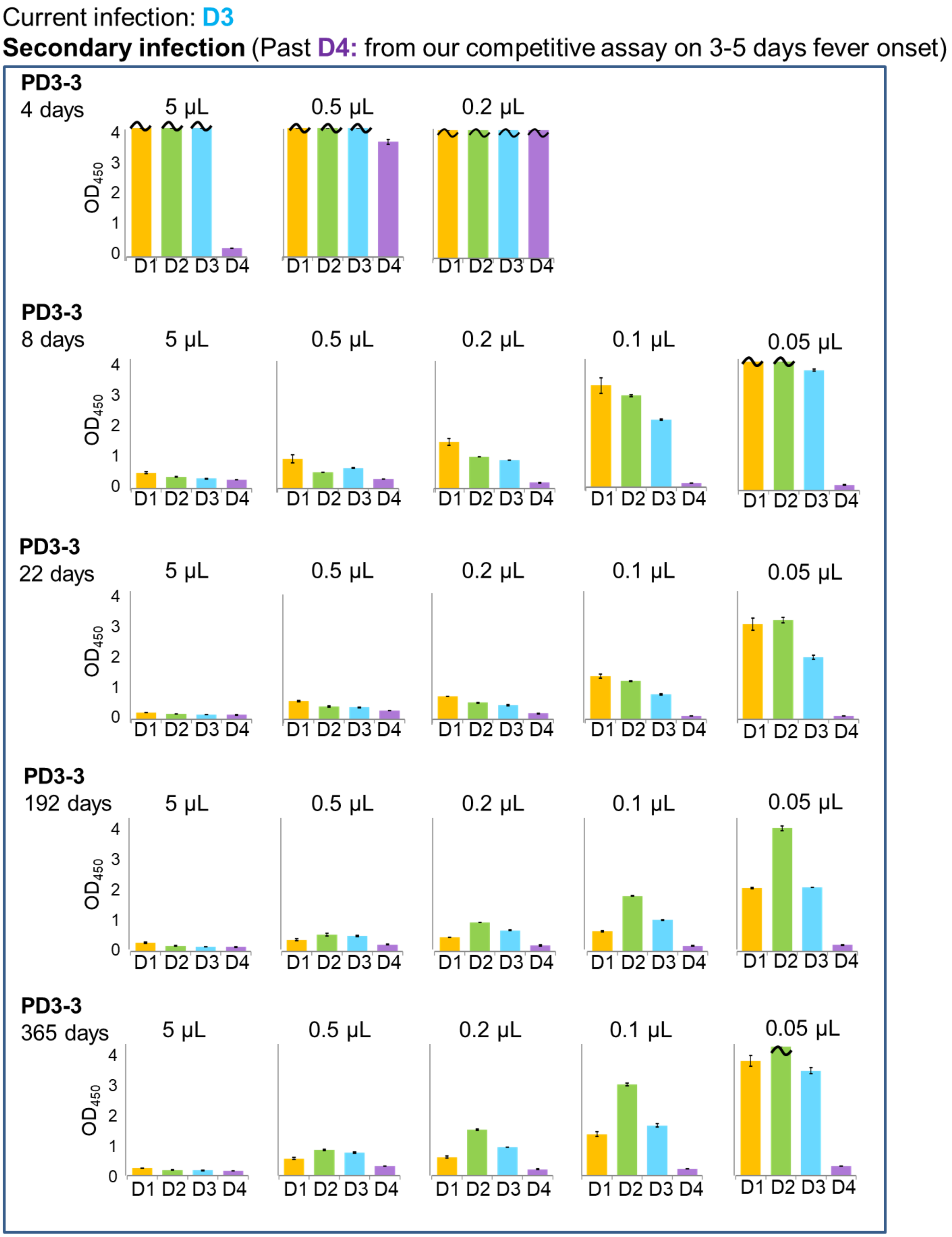

Supplementary Figure 5. Competitive ELISA using Apt/Ab pair for the patient sample, PD3-3. Anti-DEN-NS1 IgG activities were quantified from these data and summarized in Figure 4.

Current infection: D3

Primary infection (from our competitive assay on 3-5 days fever onset)

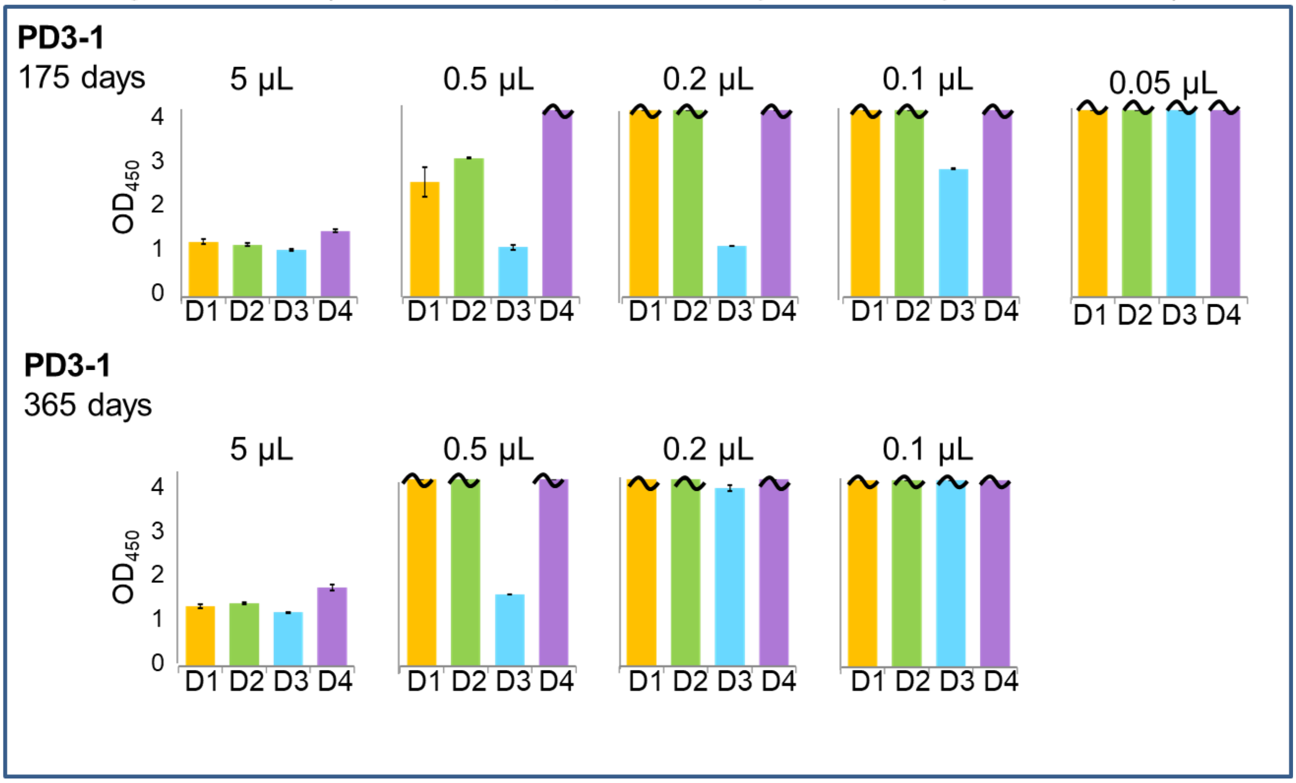

Current infection: D4

Primary infection (from our competitive assay on 3-5 days fever onset)

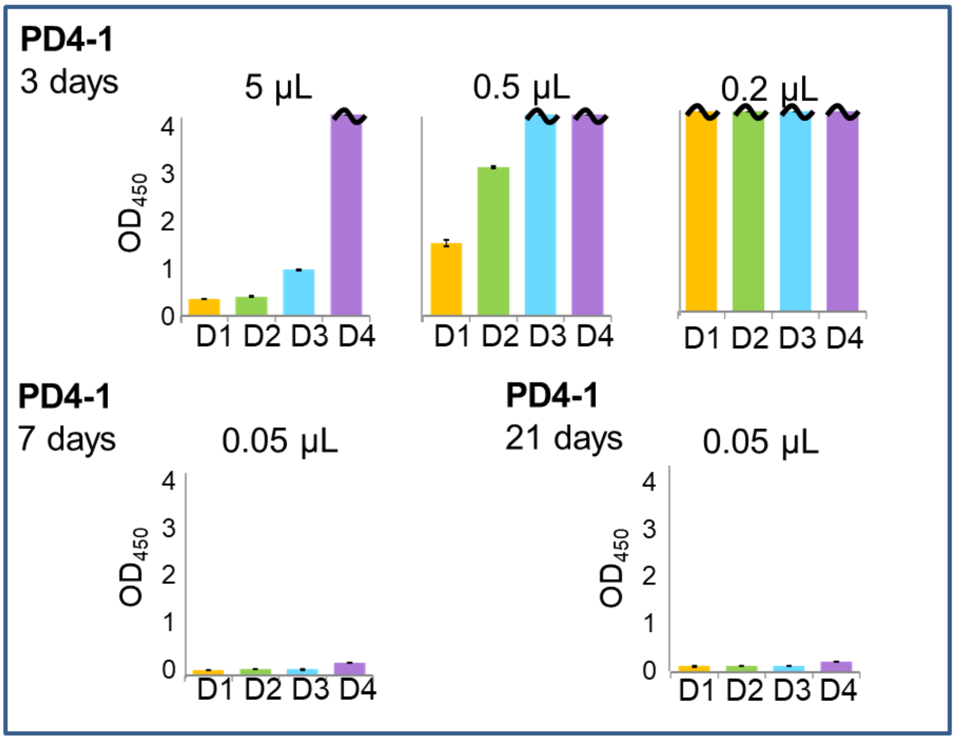

Supplementary Figure 6. Competitive ELISA using Apt/Ab pair for the patient samples, PD3-1 and PD4-1. Anti-DEN-NS1 IgG activities were quantified from these data and summarized in Figure 4.

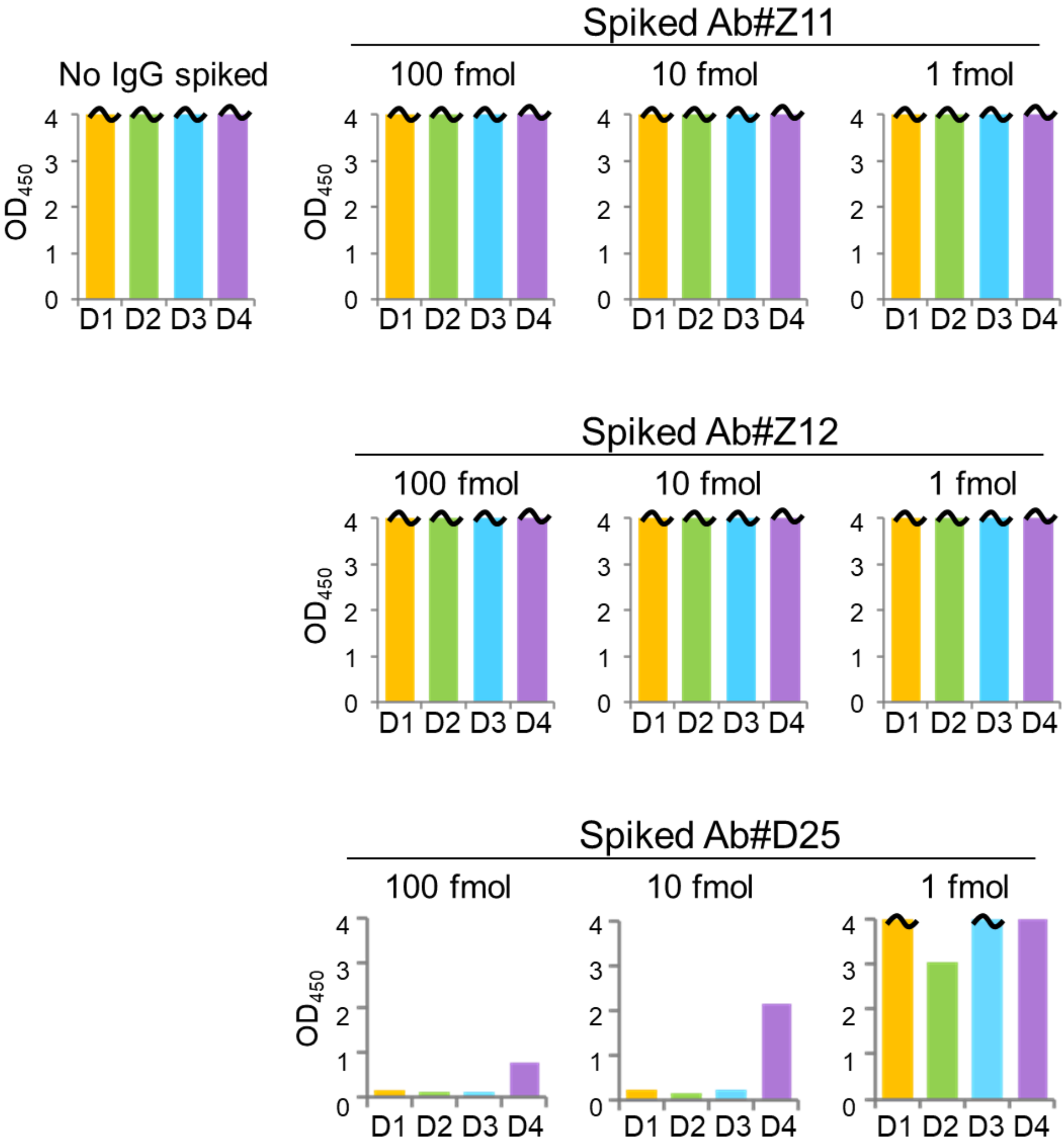

Supplementary Figure 7. Competitive ELISA using Apt/Ab pair for the control samples spiked with monoclonal antibodies against ZIKV and DENV NS1 proteins. Control human serum samples (PD0-1, 4.5 μL) were mixed with different amounts of several anti-NS1 IgGs (0.5 μL of 200, 20, or 2 nM rabbit monoclonal antibodies: Ab#Z11 and Ab#Z12 to ZIKV NS1 proteins and Ab#D25 to DENV NS1 proteins), and the mixtures, mimicking patient serums, were subjected to our competitive ELISA using the Apt/Ab pair. Ab#Z11 and Ab#Z12 are monoclonal antibodies generated through rabbit immunization with a recombinant ZIKV NS1 protein, and they are specific ZIKV NS1 binders (*K*d values: 9~15 pM for Ab#Z11 and 61~77 pM for Ab#Z12), but do not bind to DENV NS1. In our ELISA system using a sandwich Ab/Ab pair, Ab#D25 is the capture agent and Ab#D06 is the primary detector agent (see Figure 7a). We also found that DEN-NS1 binding by AptD1, AptD2, AptD3, and AptD4 is inhibited in the presence of Ab#D06, by gel-mobility shift assays (data not shown).

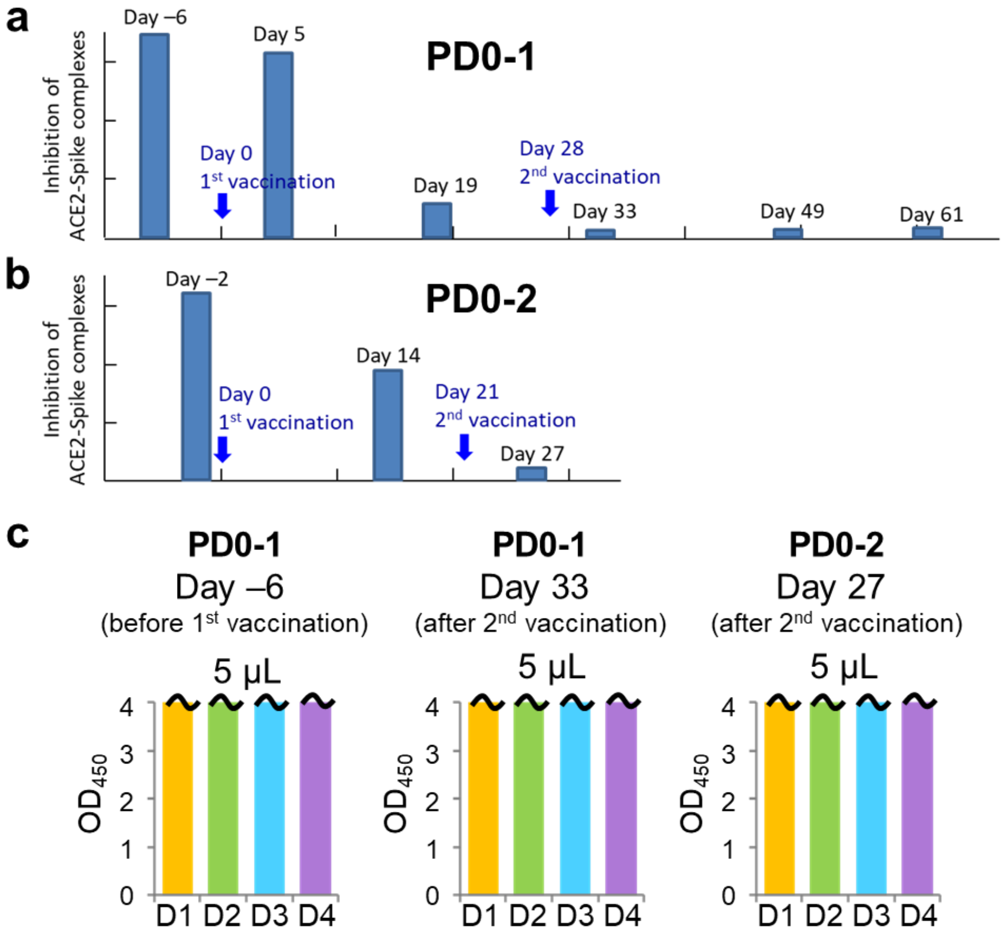

Supplementary Figure 8. Competitive ELISA using Apt/Ab pair for the COVID-19 vaccinated samples. a and **b.** Detection of total SARS-CoV2 neutralizing antibodies in control human serums (a: PD0-1, b: PD0-2), using a GenScript cPass kit (SARS-CoV2 Surrogate Virus Neutralization Kit), before and after COVID-19 vaccination with the Pfizer-BioNTech COVID-19 mRNA-based vaccine. **c.** No competitive IgGs cross-reactive to dengue NS1 were detected by our competitive ELISA format.
